# A word order typology of adnominal person

**DOI:** 10.1515/lingty-2023-0080

**Published:** 2024-11-04

**Authors:** Georg F.K. Höhn

**Affiliations:** Georg-August-University Göttingen, Göttingen, Germany

**Keywords:** adnominal pronoun constructions, pronominal determiners, headedness, demonstratives

## Abstract

This paper investigates cross-linguistic variation in the expression of adnominal person (pers_n_; cf. English “we linguists”) based on a survey of 114 languages, focusing on word order. Two subtypes are distinguished according to whether pers_n_ is expressed by an independent pronoun as in English or by a morphologically dependent marker. Prenominal adnominal pronouns are the most common type of pers_n_ marking overall, while the morphologically dependent markers are predominantly postnominal (or phrase-final). The order of pers_n_ marking relative to its accompanying noun is shown to interact with head-directionality (VO/OV-order, position of dependent genitives, adpositions) and with the position of demonstrative modifiers (prenominal/postnominal) using generalised linear mixed-effects models. Theoretical implications and possible explanations for deviations are discussed concerning variation in the encoding of pers_n_ as head or phrasal modifier and its (lack of) co-categoriality with demonstratives.

## 1 Introduction

Research on grammatical person has largely focused on the nature of person features, especially based on pronominal systems (e.g. Ackema and Neeleman 2018; Cysouw 2002; Déchaine and Wiltschko 2002; Harbour 2016; Harley and Ritter 2002; Nevins 2007, 2011), on verbal agreement (e.g. Corbett 2006), or both (e.g. Cysouw 2003, 2005; Siewierska 2004). This paper presents aspects of a first larger scale investigation of cross-linguistic variation in the expression of adnominal person (pers_n_), the morphological marking of grammatical person in nominal expressions indicating how discourse participants relate to their reference set, i.e. whether the author and/or addressee of an utterance or neither is included. English *we linguists*, for instance, denotes a set of linguists including the author. pers_n _marking may, however, also involve third person (e.g. Bernstein 2008; Longobardi 2008; Louagie and Verstraete 2015).

This paper draws on a survey of pers_n_ marking in 114 languages (the analysis comprises a sample of 113 languages), focusing on the position of pers_n_ marking relative to the head noun. While pers_n_ marking is prenominal in most surveyed languages, postnominal marking is also attested; see (1) from the Trans-New Guinea (TNG) language Amele.1Glossing in examples is adapted to conform to a common standard based on the Leipzig Glossing Rules (https://www.eva.mpg.de/lingua/resources/glossing-rules.php). Brackets and bold-face may be added to highlight the pers_n_ construction. Language names are indicated whenever the language represented differs from the directly preceding example.

(1)

**Table:** 

Amele				
*[Dana*	*ben*	*eu*	***age*** *]*	*ho-ig-a.*
man	big	that	3pl	come-3pl-todpst
‘Those leaders (big men) came.’				
(after Roberts 1987: 210, (283)–(284))				

The paper’s aims are twofold. Descriptively, it provides a first larger-scale survey of word order patterns for pers_n_, specifically the pre- or postnominal positioning of pers_n_ marking. From a theoretical perspective, it investigates the hypothesis that cross-linguistic variation in the (pre-/postnominal) directionality of pers_n_ marking shows a tendency to pattern with two other word order-related properties: head-directionality (specifically the order of adpositions, genitive modifiers, object-verb order) and the directionality of demonstrative modifiers. The observations are in line with the hypotheses that (a) pers_n_ cross-linguistically tends to show head-like behaviour and (b) demonstratives and pers_n_ tend to behave as members of the same distributional category. However, these are statistical universals, suggesting that two points of cross-linguistic variation (i.e. potential parameters) are involved.

Because of restricted data availability I do not address the interaction of pers_n_ marking with adjectives or numerals.2There may be a tendency for pers_n_ marking to occur at the left or right periphery of nominal expressions, cf. also Louagie and Verstraete (2015), maybe resembling the peripheral expression of verbal subject (person) agreement (Julien 2002: 249f.). However, see Choi (2014: 151f.) for the possibility of adjective–pronoun–noun order in Korean. Moreover, I focus on argumental uses of pers_n_-marked phrases and will not address exclamative expressions like *you idiot!* here. While they are often similar or identical to argumental pers_n_-marked expressions and have led to insightful discussion (e.g. Bernstein 2008; Julien 2016; Kluge 2017: 358f.), their behaviour can differ from argumental uses in at least some languages (see Corver 2008: 52–55). Another phenomenon put aside here are associative constructions or inclusory pronouns (Holmberg and Kurki 2019; Lichtenberk 2000; Sigurðsson and Wood 2020). They involve non-singular pronouns in construction with a nominal expression denoting a proper subset of the referents of the complete, potentially non-homogeneous expression (i.e. the equivalent of *they John* denotes “John and his associates”, not a group of Johns). The nominal description in pers_n_, on the other hand, exhaustively and homogeneously characterises the members of the denoted group, i.e. *you linguists* denotes a group of linguists including the addressee(s), not an addressee (who may not be a linguist) and a group of linguists.

This paper is structured as follows. Section 2 introduces terminological and theoretical background and presents the hypotheses under investigation. Section 3 outlines the language survey. Section 4 summarises the empirical results and presents a statistical analysis of the patterns of interest. The theoretical significance of the results and deviations from the hypotheses is discussed in Section 5. Section 6 concludes the paper.

## 2 Theoretical background

This section introduces further terminology for pers_n_ marking (Section 2.1), presents theoretical background alongside the investigated hypotheses (Section 2.2) and delineates the most common subtype of pers_n_, adnominal pronoun constructions (APCs), from similar constructions (Section 2.3).

### 2.1 Terminology

The overall phenomenon under discussion can be defined as in (2). It is understood to be distinct from the cross-referencing of person features on arguments in verbal morphology and from nominal morphology indicating the person of a possessor.

(2)

**Table:** 

**Adnominal person (** **pers** _**n**_ **)**
morphological encoding of the grammatical person of a nominal expression indicating the relation of its reference set to the discourse participants, i.e. does it include the author and/or addressee of an utterance or neither

While unaccompanied/“plain” personal pronouns may also encode pers_n_ as part of an extended nominal projection (*x*nP) (Grimshaw 2005; Panagiotidis 2015; van Riemsdijk 1998), the focus here is on pers_n_ marking of nominal expressions containing overt lexical material (nouns, adjectives). I distinguish two types of overt pers_n_ marking based on its morphological realisation.3Some (but not all) consistent null subject languages allow non-pronominal subjects to co-occur with non-third person verbal agreement, e.g. (i). This so-called unagreement (Hurtado 1985) may involve covert pers_n_ marking (Choi 2014; Höhn 2016; Höhn et al. 2017). For alternative analyses see, e.g. Ackema and Neeleman (2013, 2018) and Taraldsen (2021).

(i)

**Table:** 

Spanish				
*[Las*	*mujeres]*	*denunciamos*	*las*	*injusticias.*
det.pl	women	denounced.1pl	the	injustices
‘We women denounced the injustices.’				
(after Hurtado 1985: 187, (1))

The most commonly attested type are APCs as defined in (3) and exemplified by English *we linguists*, where a pronoun forms a close grammatical unit with the remainder of a nominal expression. The term APC is adapted from Rauh (2003), but the phenomenon is also referred to as pronoun-noun or noun-pronoun construction (Choi 2014; Comrie and Smith 1977; Patz 2002), pronominal determiner (e.g. Roehrs 2005) or, particularly with some Papuan languages, pronominal copy (Davies 1989: 107f.; Reesink 1987: 53f., 167; Roberts 1987: 162; Scott 1978: 100).

(3)

**Table:** 

**Adnominal pronoun construction (APC)**
overt pers_n_ marking of a nominal expression/*x*nP containing non-pronominal material (e.g. a noun or adjective) by means of a subconstituent that can also occur as an independent personal pronoun

The second type of overt pers_n_ marking is the bound person construction (BPC) characterised in (4). BPCs are found in a smaller number of languages, with the marking typically occurring at the edge of nominal phrases as illustrated by the first person singular marker *-na:* in (5) from the TNG language Fore.

(4)

**Table:** 

**Bound person construction (BPC)**
overt pers_n_ marking of a nominal expression/*x*nP containing non-pronominal material (e.g. a noun or adjective) by means of a phonologically bound marker

(5)

**Table:** 

Fore		
*[aogi*	*yagara:’-* ***na:*** *]*	*kana-u-e*
good	man-1sg	come-1sg-ind
‘I, the good man, come.’		
(after Scott 1978: 80)		

Bound pers_n_ markers are found under various designations in the literature, including pronominal prefix (Gravelle 2010), person prefix (Andrews 1975), nominal designant (Haacke 1977), pronominal apposition (Haiman 1980), appositional pronoun (Scott 1978), affixed form of the personal pronoun (Renck 1975), pronominal enclitic (Obata 2003), person/number/gender clitic (Whitehead 2006) or PGN/person-number-gender (PNG)-marker/suffix (Bruce 1984; Haacke 2013; Kilian-Hatz 2008).

Personal pronouns (exponents of pers_n_ in present terminology) and demonstratives have been argued to form one distributional category (Blake 2001; Choi 2014; Jenks and Konate 2022; Lyons 1999; Rauh 2003; Sommerstein 1972) because they both involve deixis (or indexation, cf. Jenks and Konate 2022) and they are in complementary distribution in many languages. However, some languages allow their co-occurrence in pers_n_/pronoun-demonstrative constructions (PPDCs) as defined in (6) and illustrated in (7), suggesting cross-linguistic variation concerning whether person and demonstrative features are encoded jointly (Höhn 2015, 2017: ch. 7; Hsu and Syed 2020).

(6)

**Table:** 

**Pers** _ **n** _ **/pronoun-demonstrative construction (PPDC)**
a nominal expression/*x*nP simultaneously containing distinct pers_n_ and demonstrative markers

(7)

**Table:** 

Manambu						
*[**də***	***a-də***	*nəkə-də*	*du]*	*ata*	*ya:d*	*kwarba:r*
he	dem.dist-m.sg	other-m.sg	man	then	go.3m.sg	bush.lnk.all
‘That very one (previously mentioned important participant) other man then went to the bush.’						
(after Aikhenvald 2008: 198, (10.3))						

### 2.2 Previous work and hypotheses under investigation

Most pers_n_-related work has focused on APCs in individual languages like Basque (Artiagoitia 2012), English (Delorme and Dougherty 1972; Keizer 2016; Panagiotidis 2002; Pesetsky 1978; Postal 1969; Sommerstein 1972), German (Lawrenz 1993: ch. 6; Rauh 2003, 2004; Roehrs 2005), Hoava (Palmer 2017), varieties of Italian (Cardinaletti 1994; Höhn et al. 2016), Japanese (Furuya 2008; Inokuma 2009; Noguchi 1997), Papuan Malay (Kluge 2017: ch. 6.2), Romanian (Cornilescu and Nicolae 2014) or Warlpiri (Hale 1973).

A few publications offer a wider cross-linguistic perspective. Arguing against an appositive analysis for English APCs, Pesetsky (1978) presents some data from Russian, French and German (with additional languages mentioned without examples). Himmelmann (1997: 213–219) and Lyons (1999: 141–145) address pers_n_-related data from several languages (e.g. Khoekhoe/Nama) when discussing whether pers_n_ is (Lyons 1999) or is not (Himmelmann 1997) related to articles and definiteness, but provide no detailed description of a larger sample. Based on contrasts between standard Italian and Greek as well as some additional languages, Choi (2014) and Höhn (2016) independently argue that the availability of the unagreement phenomenon from footnote 3 is related to properties of nominal structure, specifically the occurrence of definite articles in APCs. APCs and their relation to nominal determination in several Australian languages are addressed in a handout by Stirling and Baker (2007) and in a detailed typological study focusing on third person APCs by Louagie and Verstraete (2015) (cf. also Louagie 2017: ch. 5). Based on a wider language sample Höhn (2020) observes that the lack of third person APCs in languages like standard English (*they linguists) is largely restricted to Europe and adjacent areas and typically implies the existence of definite articles.

This is expected if definite articles in such languages are allomorphs of third person pronouns as proposed by the pronominal determiner analysis, a prominent approach to APCs in languages like English or German (Abney 1987; Lyons 1999; Postal 1969; Rauh 2003; Roehrs 2005 among others). Based on the complementary distribution of adnominal pronouns and definite articles in the relevant languages, this analysis holds that both compete for realisation of the same syntactic head D in (8). Some further arguments in favour of this approach are sketched in Section 2.3.2 below.

(8)

**Table:**
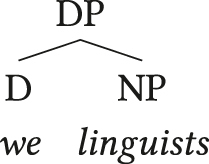

As mentioned earlier, there are languages with obligatory definite articles in APCs (Choi 2014; Höhn 2016), so an analysis building on the complementary distribution of adnominal pronouns and definite articles cannot be universally applicable. However, I adopt from the pronominal determiner analysis the generalised working hypothesis that pers_n_ marking corresponds to a syntactic head in the *x*nP in a substantial number of languages. This admits the possibility that pers_n_ marking can also be marked by non-heads (e.g. specifiers, phrasal adjuncts) in some languages.4Sybesma and Sio (2008) suggest that demonstratives cross-linguistically vary between being specifiers and heads and given their potential similarity to personal pronouns discussed earlier pronouns may plausibly also vary in this respect. The range of variation implied here is illustrated in the decision tree in (9), modelled after the parameter hierarchies of Roberts (2012, 2019) and related work. The rightmost leaf of the tree is a shorthand for various other potential syntactic positions of pers_n_ heads within the *x*nP.5This may involve variation concerning which grammatical features cluster together, cf. Giorgi and Pianesi (1997), Hsu and Syed (2020), and Höhn (2017).

(9)

**Table:**
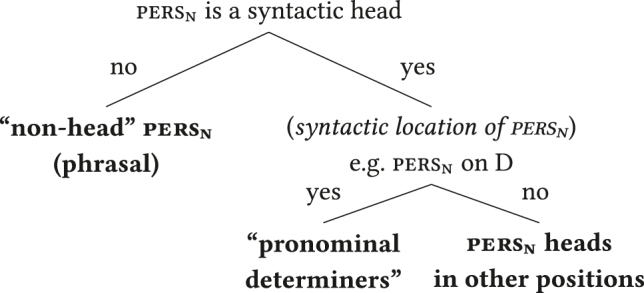

Word order patterns have been argued to show systematic cross-linguistic correlations across different domains (Dryer 1992, 2009, 2011; Greenberg 1963; Hawkins 1980, 1983), among them verb-object order (VO vs. OV), adposition directionality (pre- vs. postpositions) and the order of dependent genitives (NGen vs. GenN). One theoretical perspective on such correlations is a head-directionality parameter (Huang 1982; Koopman 1984; Stowell 1981; Travis 1984) which treats these correlating configurations as instances of head-initial (VO, prepositions, NGen) or head-final (OV, postpositions, GenN) structures respectively. However, one language-wide parameter simply set to either head-inital or head-final is now widely agreed to be too strong, since these correlations are not deterministic. Nevertheless, there are still suggestive tendencies for cross-categorial harmony in word order patterns (Baker 2008; Greenberg 1963; Hawkins 1980, 1982, 1983), such that, for instance, a language with postpositions (head-final PPs) is more likely to display OV order (head-final VPs) than not. I will not attempt an explanation here, but for recent theoretical approaches to the relationship between macro- and micro-parametric variation in terms of learning mechanisms and the concept of (emergent) parameter hierarchies see, e.g., Baker (2008), Roberts and Holmberg (2010), Biberauer and Sheehan (2013), Biberauer and Roberts (2016), Roberts (2019) and van der Wal (2020).

If a substantial number of languages treat pers_n_ as a syntactic head in the *x*nP (answering “yes” to the topmost question in (9)), its directionality relative to the remaining nominal expression and specifically the lexical noun is expected to display a cross-linguistic tendency to pattern harmonically as formulated in Hypothesis 1 (10).

(10)

**Table:** 

**Hypothesis 1** (pers_n_-as-head and harmonic head-complement order)
Assuming that pers_n_ commonly behaves as syntactic head, the directionality of pers_n_ marking tends to match that of other syntactic heads in a language, specifically that of verbs relative to objects, adpositions to their complements or nouns relative to dependent genitives.

At a reviewer’s request, I also briefly address the relation to the directionality of definite articles. If definite articles also behave as syntactic heads, Hypothesis 1 applies, predicting a tendency to match in directionality with pers_n_ marking. For the subclass of languages where definite articles cannot occur in APCs the pronominal determiner analysis predicts an even stronger effect, see Hypothesis 1a (11). Adnominal pronouns and definite articles should match directionality not only due to (violable) harmony effects, but because they occupy the same position in the relevant languages.

(11)

**Table:** 

**Hypothesis 1a** (Word order of pronominal determiners)
The directionality of adnominal pronouns and definite articles relative to the lexical noun should match in languages where definite articles are in complementary distribution with APCs.

Another issue beyond headedness concerns the aforementioned proposal that personal pronouns and demonstratives form a single distributional category, based on their similar deictic semantics and their complementary distribution in many languages (Blake 2001; Choi 2014; Lyons 1999; Rauh 2003; Sommerstein 1972). Jenks and Konate (2022) capture this in the single index constraint (SIC). The existence of PPDCs (Section 2.1) raises some challenges for a universal statement to this effect (see Section 5.3), but it seems a plausible working assumption that the cited literature is indicative of a cross-linguistic tendency for demonstratives and pers_n_ to be members of the same distributional category.6This may be parametrised similar to (9). Such co-categoriality also entails a structural parallel between pers_n_ and demonstrative modifiers. If this is cross-linguistically common, Hypothesis 2 in (12) should hold.

(12)

**Table:** 

**Hypothesis 2** (Co-categoriality of pers_n_ and DEM)
The directionality of pers_n_ marking tends to match the directionality of demonstrative modifiers.

### 2.3 Delineating APCs

APCs can resemble other configurations involving independent personal pronouns, such as resumptive pronouns that happen to be adjacent to a co-referent noun phrase or appositions. Below, I summarise arguments that APCs cannot be subsumed under these phenomena. Section 2.3.2 specifically presents some of the arguments in favour of a pronominal determiner analysis for languages like English.

#### 2.3.1 APCs and resumptive pronouns

Prenominal APCs are easily distinguished from resumptive pronouns that happen to be directly adjacent to the noun phrase they refer to because resumptive pronouns would occur only after the noun phrase, in contrast to the prenominal pers_n_ marker. Indeed, the fact that the non-pronominal part of a (non-third) pers_n_-marked phrase is not independently accessible by third/default person resumptives, with the resumptive pronoun instead agreeing in person with the adnominal pronoun as shown in (13), further supports the view that the whole pers_n_ expression forms a syntactic unit.

(13)

**Table:** 

*[You teachers]*_*i*_ *, {* ***you***_***i***_ /* ***they***_***i***_ *} have a tough job.*

Postnominal APCs, on the other hand, can be string-identical to configurations where a bare nominal happens to be followed by a resumptive pronoun. However, several empirical arguments suggest that a distinct class of postnominal APCs exists. The crucial point of differentiation is that in APCs the pronoun is a co-constituent of the associated nominal expression, while a resumptive pronoun is not an immediate co-constituent of the noun phrase it is co-referent with.

Kluge (2017: 358f.) argues in detail that Papuan Malay pronouns can form constituents with a preceding nominal expression, corresponding to postnominal APCs. Among other arguments, she shows that postnominal APCs occur in contexts where the pronoun cannot be construed as a resumptive. For instance, postnominal pronouns as part of isolated exclamatives (*babi ko* pig 2sg ‘you pig’; Kluge 2017: 353, (62)) or in clause-final position (14a) lack further clausal structure that the postnominal pronoun could act as a resumptive in. Additionally, in (14a) the final demonstrative “[has] scope over the pronoun[s]” (Kluge 2017: 358) meaning that the pronoun is embedded in a larger nominal structure. This also holds in (14b), which additionally contains a resumptive pronoun following the whole nominal complex. Kluge (2017: 359) also observes that speakers include the postnominal pronoun when repeating a nominal phrase after hesitating (14c), suggesting that the APC is perceived as a unit.

(14)

**Table:** 

Papuan Malay

a.

**Table:** 

*[Wili*	***ko*** *]*	*jangang*	*gara*	*gara*	*[tanta*	***dia***	*itu]!*
Wili	2sg	neg.imp	redup	irritate	aunt	3sg	dem.dist
‘you Wili don’t irritate that aunt!’							
(Kluge 2017: 354, (69))

b.

**Table:** 

*[Barce*	***ko***	*ini]*	*[ko]*	*takut*
Barce	2sg	dem.prox	2sg	feel.afraid(.of)
‘you Barce here, you feel afraid’ (Kluge 2017: 358, (83))

c.

**Table:** 

*[pace*	***de*** *],*	*[pace*	***de*** *]*	*mandi*	*rapi,*	*de*	*mandi*	*rapi*
man	3sg	man	3sg	bathe	be.neat	3sg	bathe	be.neat
‘the man, the man bathed neatly, he bathed neatly’								
(Kluge 2017: 359, (86))

Evidence supporting the constituenthood of postnominal APCs can also be found in a construction in the TNG language Amele where in (15) “the nominal or NP is in apposition to the [first; GFKH] pronoun and is separated from the [prenominal; GFKH] pronoun by a slight pause and has its own intonational peak” (Roberts 1987: 210), while being optionally followed by another pronoun.7Roberts indicates a pitch upstep on *dana* ‘man’ followed by a fall on the second *age* ‘3pl’. According to Roberts (1987: 210), the intonation suggests a closer relationship of the head noun to that postnominal pronoun than to the appositive prenominal pronoun.8Comparable data have been used to argue that English-style APCs are not appositions. Expressions like *[we], (that is) [we linguists]* are non-tautological, indicating that the syntactic relationship between *[we]* and *[we linguists]* is different from that internal to *[we linguists]*, cf. Lawrenz (1993: ch. 6).

(15)

**Table:** 

Amele				
*Age,*	*[dana*	*(* ***age*** *)],*	*na*	*qete-ig-a.*
3pl	man	(3pl)	tree	cut-3pl-todpst
‘They, the men, chopped down the tree.’				
(after Roberts 1987: 210, (282))				

Moreover, Roberts (1987: 217) argues that the double occurrence of the third person pronoun in (16) suggests that the first pronoun is not a resumptive and instead belongs to the subject phrase, cf. the Papuan Malay example (14) above. This supports the notion that postnominal pronouns can be subconstituents of complex nominal expressions in Amele.

(16)

**Table:** 

*[Dana*	*i/eu*	***age*** *]*	*age*	*Hilu*	*dec.*
man	dem.prox/dem.dist	3pl	3pl		from
‘These/those men are from Hilu.’					
(after Roberts 1987: 217, (315))					

Terrill (2003: 171) suggests that postnominal pronouns in the Solomon Islands isolate Lavukaleve “function resumptively”, but also notes that in (17a) “*malav e* functions as an external topic, thus lit: ‘and we people, no-one died’ ” (p. 172). A resumptive pronoun normally acts as an independent core argument of the clause, but the subject position/function of the clause is already occupied by *roa-ru* ‘no one’ here. Therefore, Terrill’s analysis of *e* ‘we’ as a subconstituent of the external topic, corresponding to a postnominal APC, seems more plausible than a resumptive analysis. In (17b), the marked APC seems to be an afterthought, further supporting its constituenthood and a non-resumptive analysis for the postnominal pronoun, since there is no following core clause in which it could function as a resumptive (cf. also the discussion by Kluge 2017: 358f. regarding Papuan Malay mentioned earlier).

(17)

**Table:** 

Lavukaleve

a.

**Table:** 

*aka*	*[malav*	***e*** *]*	*roa-ru*	*kiu-la-m.*
then	people	1pl.excl	one.sgm-none	die-neg-sgm
‘And we, the people [lit: the people we] didn’t die. [i.e. None of us people died.]’				
(after Terrill 2003: 171, (197))

b.

**Table:** 

*e-tairi-re,*	*foiga*	*e-u.*	*[Kanege*	***e*** *-sa].*
3sg.n.obj-divide-nf	dem.sg.n	3sg.n.obj-eat	family	1pl.excl-group
‘…dividing it up, [we] eat it. My family and I.’				
(after Terrill 2004: 435, (27))

Specific postnominal occurrences of pronouns may be ambiguous between analyses as postnominal pers_n _marking or as resumptive pronouns. However, the above considerations show that postnominal APCs are distinct from resumptive pronouns that just happen to occur immediately after a co-referential noun phrase.

#### 2.3.2 APCs and apposition

Various arguments have been presented against assimilating APCs to apposition in languages like English, German or Greek (e.g. Höhn 2016, 2020; Lawrenz 1993; Lyons 1999; Pesetsky 1978; Rauh 2003; Roehrs 2005; Sommerstein 1972; Stavrou 1995). Note that diagnostics presented here may not be easily cross-linguistically applicable (e.g. when relying on definite articles or verbal agreement). Crucially, the point here is not to argue that APCs are generally pronominal determiners – in fact, Section 2.2 has pointed to wider structural variation. Rather, I aim to show that there are instances of APCs that are clearly distinct from (loose and close) apposition, motivating an independent treatment of the phenomenon.

One line of argument concerns the distinction of loose apposition (Burton-Roberts 1975) from APCs, with the latter forming a more tightly integrated grammatical unit (cf. the pronominal determiner analysis introduced earlier). A commonly mentioned difference between the two is the possibility of an intonational break between the pronoun and the appositive nominal. The availability of such a break alone does, however, not show that the collocation could not be an APC.9The relationship between appositivity and prosodic correlates is not deterministic in the domain of “restrictive” versus “appositive” relative clauses in German (Birkner 2008; Blühdorn 2007) and has not been investigated for pers_n_ to my knowledge. The difference becomes clearer using distributional evidence. I only present a few observations from the literature here; for further discussion the reader is referred to the references above.

Paraphrasing an argument by Pesetsky (1978: 355), English APCs can occur following a verbal particle (18a) just like full noun phrases, but unlike plain pronouns. Appositive structures with a pronominal anchor, on the other hand, are degraded in this position, with definite article (18b) and without (18c), suggesting that the pronouns alone as “anchor” of the apposition determines its syntactic behaviour.

(18)

a.

**Table:** 

He looked up the linguists / *us / [***us*** *linguists].*

b.

**Table:** 

*He looked up us, the political advisers of the PM.

c.

**Table:** 

*He looked up us, linguists from conviction.

Another argument by Pesetsky (1978: 354) illustrates that in contrast to APCs loose appositions can give rise to a structural ambiguity, attaching either to a complex nominal expression as a whole or only to an embedded restrictor/partitive expression. The construction *some of X… others of X* in (19a) invokes two distinct subsets of a consistent group X, here one *we*-group containing at least linguists and philosophers. On the only coherent interpretation, the appositives *linguists* and *philosophers* attach high, characterising the two distinct subsets *some/others of us*. The speaker must be a member of X, but they need not be either a linguist or a philosopher. APCs do not permit any structural ambiguity, accounting for the deviance of (19b). Here, *linguists* and *philosophers* have to be construed directly with the embedded pronoun. The speaker must be a member of both independent *we*-groups and no overarching group X is established, leading to inconsistency. This parallels the use of two distinct definite expressions as restrictors (20).

(19)

a.

**Table:** 

*[Some of us], [linguists], think that [others of us], [philosophers], are crazy.*

b.

**Table:** 

*[Some of [***us*** *linguists]] think that [others of [****us*** *philosophers]] are crazy.*
(after Pesetsky 1978: 354, (12))

(20)

**Table:** 

*[Some of [the linguists]] think that [others of [the philosophers]] are crazy.
(after Pesetsky 1978: 354, (10))

There are, moreover, fewer restrictions on appositions to pronominal anchors than on the nominal part of APCs. While strong quantifiers like *most* or *every* cannot directly modify the nominal part of an English APC (21a), they are acceptable in loose apposition (21b), see Höhn (2016: 563).

(21)

a.

**Table:** 

*[***We*** *{most students/every student} of this school] are fed up.*

b.

**Table:** 

*We, {most students/every student} of this school, are fed up.*

Similarly, most varieties of English disallow third person pronouns in APCs (22a), but a string-identical construction involving loose apposition is acceptable (22b).10Such person restrictions do not constitute a necessary condition, since languages differ in whether they restrict internal properties of pers_n_ constructions, e.g. concerning third person (Höhn 2020; Louagie and Verstraete 2015; Palmer 2017) or singular APCs (Rauh 2004).

(22)

a.

**Table:** 

*And [***they*** *reputable academics of various persuasions] agreed with my assessment.*

b.

**Table:** 

*And they, (that is) reputable academics of various persuasions, agreed with my assessment.*

Close appositions like *the poet Burns* (Burton-Roberts 1975) are normally produced as one intonation unit and in that respect more similar to typical APCs. However, several arguments have been adduced against assimilation here, too.

APCs typically do not permit a reversed order (cf. English *linguists we), while close apposition has been suggested to allow reversal (*the poet Burns*/*Burns the poet*; Burton-Roberts 1975: 402; Payne and Huddleston 2002: 447). Lekakou and Szendrői (2012: 114) show that in Greek close appositions like (23ab) the adjectival predicate can agree with either part of the apposition (*aetos* or *puli*) irrespective of order. If the nominal part of the APC in (23c) was a close appositive in the third person, one might expect the construction to be reversible and to allow flexible verbal person-number agreement either with the pronoun or the putative third person nominal complex. Instead, the pronoun must precede the nominal part and third person agreement is ruled out, suggesting that the APC behaves differently from close apposition.

(23)

**Table:** 

Standard Modern Greek

a.

**Table:** 

*O*	*aetos*	*to*	*puli*	*ine*	*megaloprepos/*	*megaloprepo.*
det.m	eagle	det.n	bird	is	majestic.m	majestic.n

b.

**Table:** 

*To*	*puli*	*o*	*aetos*	*ine*	*megaloprepo/*	*megaloprepos.*	
det.n	bird	det.m	eagle	is	majestic.n	majestic.m
‘The eagle that is a bird is majestic.’

c.

**Table:** 

*[* ***Emis***	*i*	*glosoloji]*	*piname/*	*pinane.
we	det.pl	linguists	are.hungry.1pl	are.hungry.3pl
‘We linguists are starving/hungry.’				
(after Lekakou and Szendrői 2012: 114, (12))

Another argument made by Roehrs (2005: 255) for German (adapted to English by Höhn 2016: 563) concerns the inability of attributive adjectives to intervene between the parts of a close apposition (24a), while APCs freely permit modifiers between the pronominal and the nominal part (24b).

(24)

**Table:** 

German

a.

**Table:** 

*[meine*	*Freundin]*	[(*lieb-e)	*Maria]*
my	friend.f.sg.nom	dear-f.sg.nom	Maria
‘[my friend] [(*dear) Maria]’			
(after Roehrs 2005: 255, (7a))

b.

**Table:** 

***ihr***	*freundlich-en*	*Kinder*
you.pl	friendly-nom.pl	children
‘[you [friendly children]]’		
(personal knowledge)

Forms of address like *Mr Smith* are also commonly treated as instances of close apposition (cf. also Greenberg 1963: 54). Hungarian contrasts with English in that titles/forms of address follow the proper name, e.g. *Bárány úr* ‘Mr Bárány’, while APCs occur in the same pronoun-noun order as in English (25). Irrespective of the specific analysis, this suggests that APCs cannot involve the same structure as (this class of) close apposition in both languages, thus representing a further – to my knowledge new – argument against simple assimilation of APCs to close apposition.

(25)

**Table:** 

Hungarian	
***mi***	*nyelvész-ek*
we	linguist-pl
‘we	linguists’
(András Bárány, pers. comm.)	

Keizer (2016) argues that apparent inversion of apposition, cf. (26), actually involves two different structures and that instead of a proper name close appositions may also contain a pronoun as an alternative “uniquely defining element” (Keizer 2016: 178). APCs would then represent a subtype of close apposition more akin to appositions with initial proper name like (26b) rather than those with final proper names like (26a) or (24a) above and the restriction of attributive adjectives could be argued to affect the “uniquely defining element”. Terms of address would have to form yet another subtype of close apposition distinct from that of APCs in order to address the Hungarian-English contrast above.

(26)

a.

**Table:** 

*Famous linguist Noam Chomsky gave a talk…*

b.

**Table:** 

Noam Chomsky *(the) famous linguist gave a talk…

Setting aside the proliferation of subtypes of close apposition, this approach raises the question why regular APCs in English, German or Hungarian do not normally use definite articles, while the definite article seems all but obligatory in the second part of close appositions with an initial proper name, cf. (26b).11Keizer (2016: 179) points to the English *we the people* construction, which may indeed involve close apposition, but see Höhn (2020: 1f.) for data suggesting that this construction differs from English APCs in several respects. In fact, Keizer (2016: 179, (9)) presents an additional argument for such a distinction, observing that only the expressions with definite articles occasionally occur with singular pronouns in English, e.g. *based on me the person*. Moreover, German or Hungarian have no obvious equivalent of this construction (personal knowledge and András Bárány pers. comm.).

The aforementioned restriction against third person APCs in English-type languages (or at least certain varieties of English; cf. Höhn 2020: 14, fn. 9; Keizer 2016: 180, 185) also remains puzzling on a close apposition analysis of English-type APCs, since close apposition otherwise overwhelmingly involves third person expressions. A pronominal determiner analysis, in contrast, straightforwardly captures these patterns as result of the complementary distribution of third person pronouns and definite articles in this type of language (Höhn 2020; Postal 1969; Rauh 2003).

While one might then suggest that APCs represent another distinct subclass of close apposition, I see no advantage over adopting the pronominal determiner analysis for languages of the English type. On the other hand, the intricacy of the required diagnostics illustrates a phenomenological similarity of close apposition and APCs, raising the possibility that in some languages APCs may turn out to be hard (or even impossible) to distinguish from close apposition (on Keizer’s 2016 definition) after all.12Cf. Acuña-Fariña’s (2016) proposal, even though I remain sceptical concerning their claim of such underdetermination for English APCs. This level of analytical detail can, however, be set aside for the current aim of investing general ordering properties in pers_n_ marking.

## 3 Methodological considerations

pers_n_ marking is rarely systematically discussed in grammatical descriptions. A notable exception is the *Routledge Descriptive Grammars* series based on Comrie and Smith’s (1977) questionnaire, which includes a question on the availability of “Pronoun-Noun Constructions”/APCs (their question 2.1.2.1.17, p. 40). Most grammars from this series are included in the present sample. The central criterion for the inclusion of further languages was the availability of information on pers_n_-related phenomena.

Considering the overall scarcity of such information I consider it important to maximise the number of potential instances of pers_n_ in the dataset (i.e. a high recall value), even at the danger of including cases that future research might analyse differently (i.e. a potentially lower precision value). As noted in the previous section, pers_n_ phenomena do not correspond to a single, cross-linguistically uniform syntactic structure and a wider approach provides a more comprehensive perspective.

Information on the absence of pers_n_ phenomena is even scarcer than on its presence, so this study does not offer predictions concerning which languages can or cannot express pers_n_. Instead, I focus on generalisations over the distribution of properties of pers_n_ where the phenomenon is attested. While the current sample is not typologically or genetically balanced, it represents a reasonable coverage for providing a first larger scale overview of the attested word order variation in pers_n_. Problems arising from the unbalanced nature of the sample for assessing the validity of the proposed effects are addressed in the statistical model in Section 4.3.

The present survey comprises data from 114 languages from 64 genera (full list and references in Supplementary Materials S1, S3), of which 113 languages from 63 distinct genera are included in the sample analysed below. Only two languages in the survey have been explicitly claimed to lack APCs: Hixkaryana and Basque. For Hixkaryana, Derbyshire (1979: 131) observes that “[p]ronoun-noun constructions are normally handled in separate equative sentences” like (27), suggesting that pronouns do not occur adnominally in this language (just like demonstrative modifiers, cf. Derbyshire 1979: 132). For lack of further information, Hixkaryana was excluded from the further discussion of pers_n_.

(27)

**Table:** 

Hixkaryana						
*m* *ɨ* *nayar* *ɨ*	*hor* *ɨ*	***amna***	*ntono.*	*n* *ɨ* *mno*	*hokono*	*rma*
species.of.leaf	seeking	we.excl	went	house	one.occupied.with	same.ref
***amna***						
we.excl						
‘We housebuilders went looking for leaves’						
(Derbyshire 1979: 131, (290))						

Basque is included, although Saltarelli (1988: 210) suggests that the pronoun and the lexical noun in *gu-k emakume-ok* ‘we women’ in (28) form two distinct nominal expressions because they carry separate ergative marking, while case and number normally only occur once at the right edge of every Basque noun phrase. Moreover, Artiagoitia (2012) points out that since Basque articles generally occur on the right edge of the *x*nP, so should pronominal determiners – contrary to fact as shown by the ungrammaticality of postnominal *gu* ‘we’ or *zuek* ‘you.pl’ in (29b).

(28)

**Table:** 

Basque			
*[[gu-* ***k*** *]*	*[emakume-* ***ok*** *]]*	*g-eu-re*	*eskubide-ak*
we-erg	woman-proxart.pl.erg	we-emph-gen	right-abs.pl
*errespeta*	*ditzate-la*	*eska-tzen*	*dugu*
respect	3pl.abs.aux.3pl.erg-comp	request-ipfv	3sg.abs.aux.**1** **pl** **.** **erg**
‘We women request that they have respect for our rights.’			
(after Saltarelli 1988: 210, (978))

(29)

a.

**Table:** 

English: ***we*** *tradesmen* / ***you*** *idiots*	(after Abney 1987: 282)

b.

**Table:** 

Basque: *merkatari ***gu*** / *tentel ***zuek***	(after Artiagoitia 2012: 32, (26))

While Basque does not have (mirrored) English-style APCs, building on Artiagoitia (2012: sec. 5) I consider the so-called proximate (Areta 2009: 67; Trask 2003: 122) or inclusive (de Rijk 2008: 501) plural marker *-ok*, also featured in (28) and typically treated as a special form of the “plain” plural determiner *-ak*, to express pers_n_ features in the Basque determiner position after all. The examples in (30) show the obligatory13There seems to be a dialectal continuum with *-ok* being used most pervasively in western varieties (Areta 2009: 67; Trask 2003: 122), while it is obligatory only for first person uses in central varieties (author’s unpublished research) and presumably unattested in eastern varieties. use of *-ok* on noun phrases cross-referenced by second and first person agreement on the auxiliary.14Glossing added. De Rijk (2008: 502) additionally describes an anaphoric/demonstrative use of the proximate marker, compare also Trask’s (2003: 122) translations of *gizon-****ok*** as ‘we men’, ‘you men’ and ‘the men here’. Against this background, I classify Basque as having postnominal BPCs. I briefly return to Basque in Section 5.3.

(30)

**Table:** 

Basque

a.

**Table:** 

*Galdu*	*didazue*			*[aita-seme-* ***ok*** *]*
spoil	3sg.abs.aux.1sg.dat.**2** **pl** . **erg**			father-son-proxart.pl.erg
*afari-ta-ko*		*gogo*	*guzti-a.*	
dinner-loc-lnk		appetite	all-det	
‘You (pl.), father and son, have spoiled my whole appetite for dinner.’				
(after de Rijk 2008: 502, (90a))

b.

**Table:** 

*Zor*	*berri-a*	*dugu*	*[euskaldun-* ***ok*** *]*	*Orixe-rekin.*
debt	new-det	3sg.abs.aux.**1** **pl** . **erg**	Basque-proxart.pl.erg	Orixe-com
‘We Basques have a new debt to Orixe.’				
(de Rijk 2008: 502, (91a))

In order to investigate the hypothesised word order correlations, the database includes information on adposition directionality (pre-/postpositions; Dryer 2013a), verb-object order (VO/VO; Dryer 2013d) and the order of dependent genitives (GenN/NGen; Dryer 2013c) as comparative indicators of head-directionality and on the directionality of demonstratives (DemN/NDem; Dryer 2013b). The data for these properties were taken from the relevant chapters in the World Atlas of Linguistic Structures (WALS; Dryer and Haspelmath 2013) where available. Missing datapoints were added manually based on grammatical descriptions. Moreover, for languages with definite articles their directionality (ArtN/NArt) and whether they occur in APCs was manually annotated in order to address Hypothesis 1a (11), as was the availability of PPDCs for the discussion of demonstratives in Section 5.3.

Concerning VO/OV-order, the present dataset deviates from Dryer (2013d) in treating German (den Besten 1977/1983; Haider 2010: ch. 1), Dutch (Koster 1975; Zwart 2011: ch. 9) and Kashmiri (Wali and Koul 1997: 54) as having basic OV (rather than a frequency-motivated VO) order. For Tuvaluan, Besnier (2000: 134) argues that “the syntactically basic word order in Tuvaluan is VSO”, so I treat it as VO. For demonstrative directionality, Dryer (2013b) marks Kokota as DemN, but the source cited (Palmer 2008: 503) clearly states that demonstratives are postposed, so it is coded as NDem.

## 4 Results

This section presents the results of the survey, potential word order tendencies and a statistical analysis of a large subset of the data to test the relevance of the proposed word order factors for understanding the directionality variation in pers_n_. Supplementary Material S1 provides the complete dataset, while Supplementary Material S3, Section 5 describes the dataset annotation and Section 6 contains glossed pers_n_ examples for each language.

### 4.1 Overview

**Figure 1:**
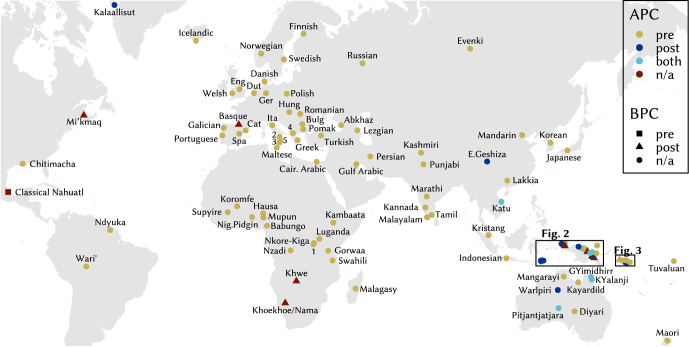
Map of APC and BPC directionality of languages included in the sample. Key: 1–Kinyarwanda, 2–Northern Calabrese, 3–Southern Calabrese, 4–Aromanian, 5–Calabrian Greek.

**Figure 2:**
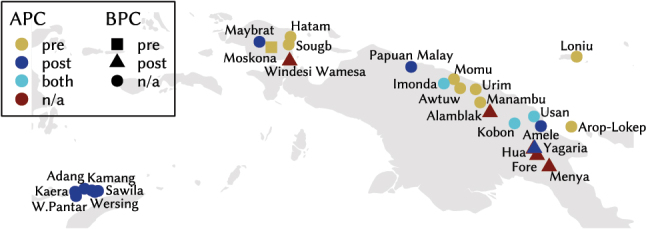
Map of APC and BPC directionality around Papua New Guinea.

**Figure 3:**
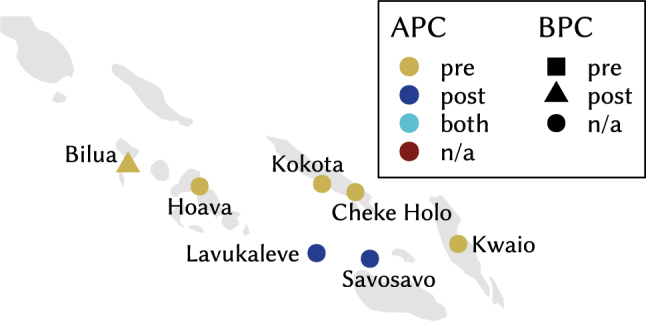
Map of APC and BPC directionality in the Solomon Islands.

Figure 1 shows the geographical distribution of APCs and BPCs as well as their respective directionality for the languages in the sample.15Maps produced using R 4.4 (R Core Team 2024) and RStudio (RStudio Team 2024) with packages mapdata (Becker et al. 2016) and scico (Pedersen and Crameri 2018) for a colour-blind appropriate palette. Language coordinates are based on Glottolog 4.5 (Hammarström et al. 2021) for most languages, on WALS (Dryer and Haspelmath 2013) for German, Norwegian and Punjabi. Coordinates correspond to Xanthi/Greece for Pomak, to Verbicaro/Italy for northern Calabrese and to Bova Marina/Italy for southern Calabrese. Colours indicate the directionality of APCs and shapes the directionality of BPCs. Circles mark languages without (evidence for) BPC and red symbols indicate languages without (evidence for) APCs. The area of Melanesia is a hotspot of variation in the type and directionality of pers_n_ in the current sample, cf. Figures 2 and 3. The clustering of postnominal APCs and BPCs there may suggest that they play a role as areal features, although they are not exclusive to this area and the property is clearly not universal even among the non-Austronesian languages of the area either.

Table 1 summarises the results with the columns indicating the number of languages with pre-, postnominal and ambidirectional APCs or BPCs.

**Table 1:** Directionality of genitives, adpositions, verb-object order and demonstratives in relation to type and directionality of pers_n_ marking. Absolute number and relative percentage within category (in italics).

	APC						BPC			
	pre		post		ambi		pre		post	
	*n*	%	*n*	%	*n*	%	*n*	%	*n*	%
Total	81		15		7		2		11	

*Genitive directionality*										

NGen	40	*49.4 %*	–		1	*14.3 %*	–		–	
GenN	33	*40.7 %*	14	*93.3 %*	4	*57.1 %*	1	*50 %*	11	*100 %*
NoDom	8	*9.9 %*	1	*6.7 %*	2	*28.6 %*	–		–	
Unclear	–		–		–		1	*50 %*	–	

*Adposition directionality*										

pre	56	*69.1 %*	2	*13.3 %*	2	*28.6 %*	1	*50 %*	1	*9.1 %*
post	20	*24.7 %*	10	*66.6 %*	4	*57.1 %*	–		9	*81.8 %*
NoDom	2	*2.5 %*	1	*6.7 %*	–		–		1	*9.1 %*
NoAdpos	2	*2.5 %*	2	*13.3 %*	–		1	*50 %*	–	
Unclear	1	*1.2 %*	–		1	*14.3 %*	–		–	

*Verb-object directionality*										

VO	55	*67.9 %*	2	*13.3 %*	–		1	*50 %*	2	*18.2 %*
OV	25	*30.9 %*	12	*80 %*	6	*85.7 %*	–		8	*72.7 %*
NC	1	*1.2 %*	1	*6.7 %*	–		1	*50 %*	1	*9.1 %*
Unclear	–		–		1	*14.3 %*	–		–	

*Demonstrative directionality*										

DemN	50	*61.7 %*	3	*20 %*	1	*14.3 %*	–		8	*72.7 %*
NDem	26	*32.1 %*	11	*72.3 %*	4	*57.1 %*	1	*50 %*	3	*27.3 %*
Mixed	4	*4.9 %*	1	*6.7 %*	2	*28.6 %*	1	*50 %*	–	
Both	1	*1.2 %*	–		–		–		–	

For each type of pers_n_ marking the lines indicate the number and relative percentage of languages with specific directionality patterns for adpositions, dependent genitives, verb-object order and demonstrative modifiers respectively.16Key for non-self-explanatory categories: NoDom – no dominant order; NoAdpos – no clear class of adpositions; mixed – both pre- and postnominal strategies available without a clear preference; both – simultaneous pre- and postnominal marking; unclear – no clear information available for this property. The sample contains 103 languages with APCs and 13 languages displaying BPCs. Note that three languages seem to allow both APCs and BPCs,17See discussion surrounding Table 3 in Supplementary Material S3, Section 1 and (40) below. so these two types of nominal person marking are not necessarily exclusive. Among APCs, prenominal pers_n_ marking is the most frequent option, while postnominal marking is more frequent among BPCs.18Supplementary Material S3, Section 1 provides some discussion of the three less common types of pers_n_ marking: postnominal and ambidirectional APCs and languages with BPCs.

### 4.2 Tendencies

Table 2 tentatively summarises word order tendencies in Table 1 above according to type and directionality of pers_n_ marking. A tendency is assumed if the most numerous value within a category has double the instances of the next lower one. For prenominal APCs, a DemN tendency is indicated in brackets because it just barely fails to meet the criterion. With the exception of object-verb order, the tendencies for ambidirectional APCs are also bracketed due to the small differences in absolute numbers. The two languages with prenominal BPCs are insufficient for establishing any tendencies.

**Table 2:** Tentative word order tendencies for different types of pers_n_ marking.

	APC_pre_	APC_post_	APC_ambi_	BPC_post_
Genitives	Not clear	GenN	(GenN)	GenN
Adpositions	Prepositions	Postpositions	(Postpositions)	Postpositions
Verb-object order	VO	OV	OV	OV
Demonstratives	(DemN)	NDem	(NDem)	DemN

Consistent with Hypothesis 1, languages with postnominal APCs and BPCs show patterns commonly associated with head-finality in all three investigated domains (postpositions, prenominal genitives and OV order), while prenominal APCs display a tendency for head-initiality at least with respect to prepositions and VO order.

Demonstrative order also suggests an interaction with pers_n_, albeit with an interesting exception. For languages with pre- and postnominal APCs demonstrative directionality patterns with pers_n_ directionality consistent with Hypothesis 2. However, postnominal BPCs display a tendency for prenominal demonstratives, resembling in this respect the (marginal) DemN tendency of prenominal APCs and contrasting with postnominal APCs.

Due to the unbalanced nature of the sample, these tendencies in the raw data have to be treated with care. The next section statistically evaluates the validity of the two main hypotheses for pre- and postnominal APCs. Further theoretical implications are discussed in Section 5, where I will also address the interaction of definite articles and APCs predicted by the pronominal determiner analysis (Hypothesis 1a).

### 4.3 Statistical analysis

The numbers of languages with BPCs and ambidirectional APCs are too small for meaningful statistical analysis, so this section exclusively focuses on pre- and postnominal APCs. A generalised linear mixed-effects model was fit using R 4.4 (R Core Team 2024) and the lme4 package (Bates et al. 2015) within the R-Studio environment (RStudio Team 2024) in order to test the significance of head-directionality (Hypothesis 1) and demonstrative order (Hypothesis 2) for the directionality of APC.19Supplementary Material S2 provides the R code.

As noted in Section 2.2, the factors adposition, genitive and object-verb order tend to correlate cross-linguistically and are not independent even conceptually due to their role as stand-ins for head-directionality in Hypothesis 1. This poses a problem for the reliability of a model directly incorporating them as predictors. At the same time, the correlations are not perfect because word order is not always harmonious, so a reduction of these factors is not trivial. I address this by deriving the factor headfin.value which is set to 0 by default and increased by 1 for every property value typically associated with “head-finality” (postpositions, GenN, OV), decreased by 1 for every “head-initial” characteristic (prepositions, NGen, VO) and left unchanged for any other value of the three relevant properties. This results in a value range between −3 for languages with three head-initial properties and 3 for languages with three head-initial properties, and values in between for languages with mixed or unclear values for some or all relevant features. Demonstrative directionality (demfin.value) is coded as 1 for languages with NDem order, −1 for languages with DemN order and 0 otherwise. This approach with the use of discrete numerical values for both predictor variables allows the inclusion of languages in the overall analysis even if their head- or demonstrative directionality value is outside the scope of the hypotheses under investigation, so the analysis can include a larger subset of the database.

The logistic regression model in (31) treats APC directionality (APCDir) as dependent variable with two predictors, headfin.value representing Hypothesis 1 and the directionality of demonstrative modifiers (demfin.value) representing Hypothesis 2. Language genus is also included as random intercept to ameliorate the impact of the unbalanced representation of language genera within the dataset.20Alternative random intercept structures including an embedded phylogenetic structure (1| family/genus) and measures of geographical distance were also considered at the suggestion of a reviewer. The model in (31) was selected as the most informative one based on the Akike information criterion (AIC). See Supplementary Material S3, Section 3 for further details on model selection.

(31)

**Table:** 

APCDir ∼ headfin.value + demfin.value + (1 | genus)

In order to allow binomial fitting, the sample was restricted to binary values for the dependent variable APCdir (prenominal/postnominal), leaving a subset of 96 languages from 51 different genera in 31 language families for the analysis. The results are presented in Table 3.

**Table 3:** Generalised linear mixed effects model on APC directionality.

	Estimate	SE	*z*	*p*	Model comparison		
					AIC_diff_	*χ* ^2^	*p*
intercept_[APCpost]_	−36.03	8.75	−4.12	< 0.001			
headfin.value_[final]_	15.57	4.34	3.59	< 0.01	−14.15	16.16	< 0.001
demfin.value_[NDem]_	24.04	6.61	3.64	< 0.01	−10.21	12.21	< 0.001

The negative estimate of the intercept reflects the higher number of prenominal APCs in the dataset. The positive estimate for headfin.value suggests that an increase in head-final characteristics is associated with an increased chance that a language displays postnominal APCs. Similarly, the positive estimate for demfin.value suggests an association between postnominal demonstratives and APCs. These results are significant at *p* < 0.001, but since the fixed effects for smaller datasets like the present one are unreliable, following Bates et al. (2015: 33f.) the log-likelihood for the maximal model in (31) was compared to that of two reduced models where one of the factors headfin.value or demfin.value is dropped respectively. The AIC is used to measure the relative goodness of fit of competing models, while penalising an increased number of parameters in order to avoid overfitting. A lower AIC value indicates a lower estimated loss of information compared to other models. The column AIC_diff_ in Table 3 lists the difference between the AIC of the full model and the smaller model without the relevant factor. The negative results indicate an increase in AIC for the reduced models compared to the maximal model in (31), suggesting that removal of either factor leads to a significant loss of informativity.

## 5 Discussion

This section discusses some theoretical implications of the results presented above.

### 5.1 Head-directionality (Hypothesis 1)

The interaction between the directionality of pers_n_ marking and head-directionality observed in Section 4 is compatible with Hypothesis 1, repeated in (32).

(32)

**Table:** 

**Hypothesis 1** (pers_n_-as-head and harmonic word order)
Assuming that pers_n_ commonly behaves as syntactic head, the directionality of pers_n_ marking tends to match that of other syntactic heads in a language, specifically that of verbs relative to objects, adpositions to their complements or nouns relative to dependent genitives.

Languages with postnominal APCs and BPCs in the sample also overwhelmingly display characteristic head-final word orders and the reverse holds for a large number of languages with prenominal APCs, as expected if pers_n_ tends to be encoded as a syntactic head in the *x*nP. This suggests that the strategy is salient enough to leave a detectable trace in the current sample. Importantly, though, it does not mean that languages universally realise pers_n_ as head in the extended nominal projection, as also suggested in (9) in Section 2.2. Against this background, the remainder of this section discusses two configurations that do not conform to the pattern predicted by Hypothesis 1: languages with ambidirectional APCs and languages with prenominal APCs and head-final word order characteristics.

The flexible positioning of person marking in languages with ambidirectional APCs (Supplementary Material S3, Section 1) is at odds with a pers_n_-as-head analysis, since head-directionality is generally assumed to be fixed for particular types of heads. As far as the pronominal determiner analysis is concerned, such word order flexibility is also untypical for article-like determiners (Himmelmann 2001: 832). While I offer no specific analyses for the languages with ambidirectional APCs here, I sketch two possible approaches. Considering the information-structural effects tentatively observed for some of these languages (Supplementary Material S3, Section 1), those might actually involve one basic APC order with the reversed surface order of noun and pronoun resulting from (information-structurally driven) movement inside the nominal domain. Moreover, as noted above adnominal pronouns – and possibly also similarly flexible demonstratives – might simply not be heads in some languages, but rather phrasal adjuncts to the noun phrase with flexible (pre- or postnominal) linearisation, similar to the flexibility observed for some English VP-adjuncts (e.g. *to [go* ***boldly****]* vs. *to [****boldly*** *go]*).

Languages with prenominal APCs form a more heterogeneous group than languages with postnominal APCs or BPCs, including a sizable number of languages with head-final characteristics. The sample contains 22 languages with prenominal APCs and at least two out of the three characteristics of head-finality considered here (prenominal genitives, postpositions, OV order); for the full list see Supplementary Material S3, Section 2.1.

On the pers_n_-as-head analysis, prenominal pers_n_ marking as indicator of head-initiality would be disharmonic with the other indicators of head-finality. This might not be too problematic, since word order harmony is widely acknowledged to be a tendency and not a universal rule after all. However, the Final-over-Final Condition (FOFC), e.g. Biberauer et al. (2014) and Sheehan et al. (2017), provides a tool to further probe structural interactions. This structural condition (33) aims to account for various structural asymmetries across languages by the assumption that head-final projections can only dominate head-final projections within the same extended projection, ruling out configurations where a head-final projection dominates a head-initial one (34).

(33)

**Table:** 

**Final-over-Final Condition** (FOFC; Holmberg 2017: 1, (1a))
A head-final phrase *α*P cannot immediately dominate a head-initial phrase *β*P, where *α* and *β* are members of the same extended projection.

(34)

**Table:**
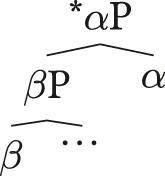

Biberauer et al. (2014: 186–189) illustrate this with Finnish. While mostly postpositional, some Finnish adpositions like *yli* ‘across’ may be used pre- or postnominally. In (35b), *yli* ‘across’ is used as a preposition taking a head-initial NP complement and FOFC imposes no restriction. In ungrammatical (35a), *yli* ‘across’ is a postposition whose complement NP is itself head-initial. If adpositions are part of the extended nominal projection at least in Finnish (Biberauer et al. 2014: 187), this configuration of a head-final PP dominating a head-initial NP corresponds to (34) ruled out by FOFC.

(35)

**Table:** 

Finnish

a.

**Table:** 

*[_*PP*_*[*_*NP*_ ***rajan***	*[*_*PP*_ *maitten*	*välillä]]*	***yli*** *]*
border	countries	between	across

b.

**Table:** 

*[*_*PP*_ ***yli***	*[*_*NP*_ ***rajan***	*[*_*PP*_ *maitten*	*välillä]]]*
across	border	countries	between
‘across the border between the countries’			
(after Biberauer et al. 2014: 187, (29))

Finnish nominal modifiers can appear prenominally using special linking morphology.21Glossed here as lnk instead of Biberauer et al.’s (2014) gloss as adjectival suffix. In (36b), the prepositional *yli* ‘across’ still trivially satisfies FOFC. However, in contrast to (35a) the head-final *yli*-PP in (36a) also causes no FOFC violation anymore, since the complex NP in its complement is now head-final as well.

(36)

a.

**Table:** 

*[*_*PP*_ *[*_*NP*_ *[*_*PP*_ *maitten*	*välinen]*	***rajan*** *]*	***yli***
countries	between.lnk	border	across

b.

**Table:** 

*[*_*PP*_ ***yli***	*[*_*NP*_ *[*_*PP*_ *maitten*	*välinen]*	***rajan*** *]*
across	countries	between.lnk	border
both: ‘across the border between the countries’			
(after Biberauer et al. 2014: 188, (31))

Twenty of the 22 largely head-final languages with prenominal APCs have postpositions (Supplementary Material S3, Section 2.1, Table 4; the others lack adpositions or clear information). If adpositions are part of the *x*nP more generally (e.g. Grimshaw 2005), a head-final PP with a head-initial (i.e. prenominal) APC complement would violate FOFC, cf. (37).22The assumption that adpositions form part of the *x*nP may be subject to cross-linguistic variation itself. Hungarian is among the 22 languages under discussion, but Höhn (2020: 26) argues that its lack of third person APCs is captured most straightforwardly if it has pronominal determiners. Postpositional phrases with APC or plain definite complements would be expected to be ruled out by FOFC, contrary to fact. This could be resolved in three ways: (a) rejecting FOFC or (b) Hungarian definite articles are not heads or (c) postpositions are not part of the *x*nP in that language. Concerning (a), I consider rejecting FOFC based on an individual datapoint premature. Concerning (b), Hungarian definite articles are widely assumed to head DP (Dékány 2011: 73; Kiss 2004: 154; Szabolcsi 1994). While I can offer no analysis here, option (c) seems to be worthy of exploration in future research. If FOFC is a general property of linguistic structure, there are different ways such languages could deal with this issue.

(37)

**Table:**
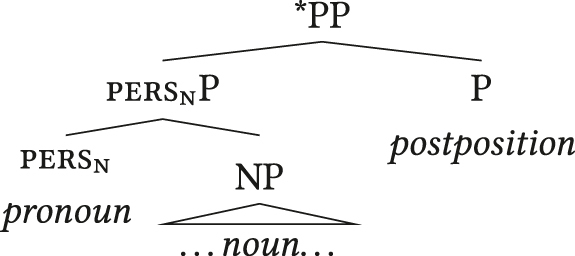

One way is to avoid FOFC-violating configurations (Biberauer et al. 2014: 179, 186–189). While I am not aware of relevant empirical data, some of these languages might avoid/disallow using postpositions with (prenominal) APC complements, which would allow maintaining a head-analysis of pers_n_ for them.

Another possibility, mentioned before with reference to the decision tree in (9), is that pers_n_ is not a syntactic head in the *x*nP in some languages of this type, so configurations of prenominal APCs with postpositions are not assigned the FOFC-violating structure in (37). Instead, pers_n_ is a phrasal modifier, for instance an adjunct to the nominal expression or a specifier of some functional projection Y as in (38).

(38)

**Table:**
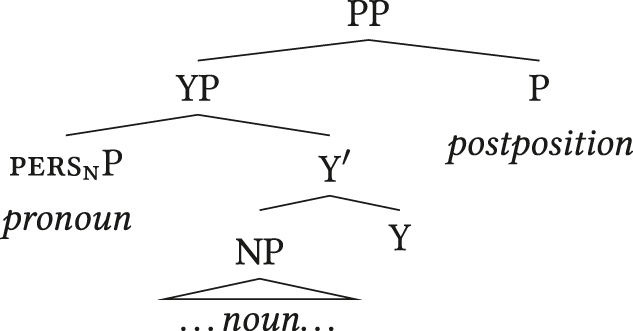

Analyses treating adnominal pronouns as specifiers have indeed been independently proposed for at least two of the 22 languages with prenominal APCs and indicators of head-finality: Finnish (Gröndahl 2015a,b; Höhn 2020: 24f.) and Japanese (Furuya 2008; Inokuma 2009; Noguchi 1997), see e.g. (39) where the pronoun *watasi-tati* ‘we’ is analysed as a phrasal DP in the specifier of a distinct DP projected over the lexical noun.

(39)

**Table:** 

Japanese				
*[*_*DP*_ *[*_*DP*_ *[*_*NP*_	***watasi-tati***	*]][*_*D’*_ *[*_*NP*_	*gengogakusya*	*]]]*
	1-pl		linguist	
‘we linguists’				
(combined from Noguchi 1997: 780, (40a) and 781, (45))				

A similar approach may apply to Bilua, one of the languages allowing both prenominal APCs and postnominal BPCs (Supplementary Material S3, Section 1), see (40a) for an example where they co-occur23The 3sg.f morpheme *ma* marks possessor phrases when the possessee is not a 3sg.m noun. The comma in the translation does not seem to be significant to the analysis. and (40b,c) for individual examples of both types. Given the tendency for head-finality across languages with postnominal BPCs (Section 4.2), I suggest that their postnominal markers realise the syntactic pers_n_ head in these languages. Prenominal pronouns in such languages could then occupy a distinct phrasal position (e.g. in the specifier of the pers_n_ head).

(40)

**Table:** 

Bilua

a.

**Table:** 

***enge*** *=a*	*Solomoni=a=ma*	*maba*	*poso=* ***ngela***
1pl.excl=lig	Solomon=lig=3sg.f	person	pl.m=**1** **pl** **.** **excl**
‘we, Solomon people’			
(Obata 2003: 85, (7.35))

b.

**Table:** 

*enge=ko*	*visi=* ***nga***
1pl.excl=3sg.f	younger.sibling=2sg
‘you who are our younger sister’	
(Obata 2003: 103, (7.116))

c.

**Table:** 

***enge*** *=a*	*saidi*
1pl.excl=lig	family
‘we, family’	
(Obata 2003: 79, (7.10))

The approach developed here offers a perspective on why prenominal APC seem to be more common than the other forms of pers_n_ marking. Prenominal APCs can be derived not only from head-initial nominal structures with pers_n_ as head, but also from configurations where pers_n _marking is introduced as a non-head, whether as specifier, adjunct or even apposition. At least specifiers have been argued to generally merge to the left to account for cross-linguistic asymmetries (Kayne 1994). Languages opting for this strategy of encoding pers_n_ would then converge on a prenominal APC irrespective of whether their *x*nPs are generally head-initial or head-final. If postnominal APCs and BPCs are largely confined to occurring in head-final pers_n_-as-head languages, while prenominal APCs can result from of a wider range of structural configurations than just head-initial *x*nPs encoding pers_n_ as head, this could at least partly explain why the latter are cross-linguistically the prevalent option.

### 5.2 Directionality of pronominal determiners (Hypothesis 1a)

This section comments on the interaction of APCs with definite articles. The sample contains 45 languages with articles, of which 41 have APCs. If not only pers_n_, but also definite articles tend to behave as syntactic heads (Abney 1987; Szabolcsi 1994), Hypothesis 1 predicts a tendency for them to match in directionality as well. This seems to be borne out, as shown in Table 4a. In languages with prenominal APCs articles tend to be prenominal and languages with postnominal APCs display the opposite tendency.

**Table 4:** Directionality of APCs and articles.

	(a) All languages with articles			(b) Languages excluding articles in APCs		
	APC_pre_	APC_post_	APC_ambi_	APC_pre_	APC_post_	APC_ambi_
**ArtN**	27	1	0	13	0	0
**NArt**	7	5	1	0	0	0

Recall that for languages with pronominal determiners Hypothesis 1a, repeated in (41), predicts an even stronger correlation between the directionality of definite articles and adnominal pronouns, since they are argued to occupy the same syntactic position.

(41)

**Table:** 

**Hypothesis 1a** (Word order for pronominal determiners)
The directionality of adnominal pronouns and definite articles relative to the lexical noun should match in languages where definite articles are in complementary distribution with APCs.

The sample contains 13 relevant languages that have articles, but exclude them in APCs. As shown in Table 4b, they all have prenominal articles and APCs in line with (41) and the predictions of the pronominal determiner analysis.

### 5.3 Demonstrative directionality (Hypothesis 2)

The findings in Section 4 concerning the interaction between the directionality of adnominal pronouns and demonstratives are consistent with Hypothesis 2 in (42).

(42)

**Table:** 

**Hypothesis 2** (Co-categoriality of pers_n_ and DEM)
The directionality of pers_n_ marking tends to match the directionality of demonstrative modifiers.

Matching directionality is expected if pronouns and demonstratives are co-categorial across a range of languages (e.g. Blake 2001; Choi 2014; Höhn 2016; Jenks and Konate 2022; Rauh 2003). It is not a sufficient criterion for categorial identity as the discussion of pers_n_/pronoun-demonstrative constructions (PPDCs) below shows, but provides a heuristic for identifying apparent or real deviations for closer inspection.

Postnominal demonstratives in most languages with postnominal APCs (Supplementary Material S3, Section 1, Table 1) are consistent with Hypothesis 2. Against the background of Section 5.1, demonstratives could occupy a final head position in the *x*nP (cf. also Sybesma and Sio 2008) with potential variation concerning whether its position is identical to that of the pers_n_ marker. The sample contains 29 languages with APCs where contrary to Hypothesis 2 the directionality of demonstratives and pers_n_ marking does not match (Supplementary Material S3, Section 2.1, Table 5).24The systematic tendency for prenominal demonstrative modifiers in languages with postnominal BPC (Section 4.2) is addressed separately below. Two potential explanations for the deviations are listed in (43).

(43)

a.

**Table:** 

The language has an additional position for demonstratives matching the directionality of pers_n_ marking as predicted by Hypothesis 2.

b.

**Table:** 

Adnominal pronouns and demonstratives do not belong to the same distributional category in the language (contra Hypothesis 2), so their directionality is not directly correlated. One indicator for this is the existence of PPDCs.

Starting with (43a), eight of these languages allow an alternative demonstrative order matching the directionality of adnominal pronouns, i.e. DemN for languages with prenominal APCs and NDem for postnominal APCs.25See the third column in S3, Table 5. The alternative word orders seem to interact with information structure. The postnominal demonstrative in East Geshiza is also analysed as a topicaliser (Honkasalo 2019: 702). Prenominal demonstratives are described as emphatic for the five Niger-Congo languages and Cheke Holo. Incidentally, Nkore-Kiga also requires the use of the emphatic pronoun set in APCs (Taylor 1985: 131). The distributional facts of these languages are then still consistent with an analysis where pronouns and (certain) demonstratives are members of the same category. Adnominal pronouns may simply have properties that restrict them to the marked position.

This also explains why Hypothesis 2 does not make clear predictions for languages classified with ‘mixed’ demonstrative order. Whatever the directionality of pers_n_ marking, there is a matching demonstrative order. Note in this context that almost all languages with ambidirectional APCs also allow pre- and postnominal demonstratives. Kuku Yalanji and Imonda are already categorised as mixed in WALS, but Pitjantjatjara (Bowe 1990: 32), Guugu Yimidhirr (Haviland 1979: 104) and Katu (Costello 1969: 33, 35) also permit the opposite demonstrative order from that marked in WALS (and Table 3 in Supplementary Material S3) in line with the assumption of co-categoriality.26Usan is the exception in that it has NDem order, but allows PPDCs (Supplementary Material S3, Section 2.2, Table 6), independently indicating that its pronouns and demonstratives are not co-categorial as discussed below.

Turning to explanation (43b) for deviations from Hypothesis 2, recall from Section 2.1 the co-occurrence of demonstratives and pers_n_ marking in pers_n_/pronoun-demonstrative constructions (PPDCs), cf. (44).

(44)

a.

**Table:** 

Cheke Holo						
*[* ***Tahati***	*naikno*	***ḡre*** *]*	*e*	*kmana*	*pui*	*puhi=da*
1pl.incl	people	dem.prox.pl	emph	lot.of	dur	way=1pl.poss
‘We people have had many problems.’						
(Boswell 2018: 165)

b.

**Table:** 

Sougb				
*Dauntoba*	*[* ***len-g***	*sogougb*	***gi-ni*** *]*	*kaba*
in.order	3pl-nmlz	slave	nmlz-dem.prox	then
*mer-uwa*	*mougb…*			
3pl-behaviour	shine			
‘In order that the slaves will be industrious…’				
(after Reesink 2002: 271, (13))

c.

**Table:** 

Koromfe			
***ʊkɔ***	*(a)*	*korombʌ*	***b*** ***ϵ*** ***ŋ***
we	art	Koromba	dem.hum.pl
‘we Koromba’			
(John Rennison, pers. comm.; cf. Rennison 1997: 251, (585))

Based on PPDCs, Höhn (2015, 2017) and Hsu and Syed (2020) argue that some languages encode pers_n_ and demonstrative features in distinct syntactic positions and thus belong to distinct distributional categories, so Hypothesis 2 is not expected to apply. Seventeen of the 29 languages with directionality mismatches display PPDCs, see Supplementary Material S3, Section 2.2, Table 5.27PPDCs are even more rarely mentioned in grammatical descriptions than pers_n_ in general, so the data are not necessarily complete.

Cheke Holo and Eastern Geshiza are treated as having an alternative demonstrative order matching pers_n_ directionality and also allowing PPDCs. In both languages different demonstratives occur in pre- and postnominal positions. The (typically) prenominal position of the marked emphatic demonstrative *u* in Cheke Holo (Boswell 2018: 169) might correspond to that of the prenominal pronoun in the PPDC in (44a), in line with Hypothesis 2.

Eastern Geshiza with its postnominal APCs and prenominal demonstratives also has an additional postnominal demonstrative *t*^*h*^ə. However, it is this marker that can occur in PPDCs, cf. (45), showing that while matching in directionality it nevertheless occupies a distinct position from adnominal pronouns. Honkasalo (2019: 301f., 702) suggests that this postnominal morpheme is developing into a topic marker and it is glossed as such in (45), so depending on the degree of grammaticalisation – i.e. whether the demonstrative and topicalising uses involve distinct morphemes – the status of (45) as PPDC may be debated. Importantly, even if one rejects that Eastern Geshiza has PPDCs on this basis, the prenominal position of the remaining demonstrative, contrasting with the postnominal pronoun, suggests that demonstratives and pronouns are not co-categorial in this language.

(45)

**Table:** 

Eastern Geshiza			
*[bæ*	***ŋæ=ɲə*** *=t* ^*h*^ *ə]*	*‘mbəzli’*	*d-ə-joŋ.*
Tibetan	1=pl=top	ritual.tripod	pref-nact-say.1
‘We Tibetans call it *mbəzli* (ritual tripod).’			
(Honkasalo 2019: 400, 5.115)			

PPDCs also occur in 18 languages with matching directionality of adnominal pronouns and demonstratives (Supplementary Material S3, Section 2.2, Table 6), suggesting that directionality is indeed not a sufficient criterion for co-categoriality. PPDCs generally represent a challenge for Jenks and Konate’s (2022) single index constraint (SIC), the proposal that there is only one referential index in nominal expressions, due to the co-occurrence of two potential referential indices in their system, pers_n_ and demonstratives. To tackle this issue, Jenks and Konate (2022: 16f., 31) propose that in languages with prenominal pers_n_ and demonstratives in PPDCs both are “packaged” in the same syntactic projection, the demonstrative heading a (referentially indexed) D_x_P and the pronoun occupying its specifier (46a). A reviewer notes that languages with PPDCs, prenominal APCs and postnominal demonstratives are consistent with Jenks and Konate’s (2022) proposal if the postnominal demonstrative heads D_x_P and hosts the adnominal pronoun in its specifier, see (46b).

(46)

a.

**Table:** 

[_DxP_ pronoun [_Dx’_ dem [_NP_ …]]]

b.

**Table:** 

[_DxP_ pronoun [_Dx’_ [_NP_ …] dem]]

This analysis might apply to at least some of these languages, but PPDCs like those in (47) are not immediately amenable to either analysis in (46). Jenks and Konate (2022: 16) justify their analysis of pronouns as specifiers with the claim that “the pronoun occurs before the demonstrative” in PPDCs, slightly oversimplifying the Extremity Of Person Hypothesis by Höhn (2017: 261, 288) suggesting that pers_n_ is typically more distant from the nominal than demonstratives. Examples (47a,b) challenge that empirical claim.28However, the grammaticality of (47a) is contested (Höhn 2015: 89f.). Following Jenks and Konate’s (2022) logic, (47a) would require treating the pronoun as head of DxP and the demonstrative as its specifier, conflicting with proposals that Japanese adnominal pronouns are specifiers (see Section 5.1). While (47b) could be derived from (46a) by movement of NP to some higher position than DxP (Abels and Neeleman 2012; Cinque 2005), the order of (46c) is the inverse of (46a) and assuming a rightward specifier-position for the pronoun contravenes the widespread assumption that specifiers generally branch to the left (but see Abels and Neeleman 2012). Moreover, for both (46b,c) any analysis building on (46) would be at odds with the proposal from Section 5.1 that postnominal APCs involve final pers_n_ heads. Finally, (46d) shows a PPDCs with three expressions of referential indices, a personal pronoun, a demonstrative and a definite (or familiarity) marker, requiring at least one additional index-related projection compared to (46), thus further challenging the SIC.

(47)

a.

**Table:** 

Japanese				
*Sensei-wa*	*[* ***sono***	***watasitati***	*gakusei]-o*	*suisensimasita.*
teacher-top	dem	1pl	student-acc	recommended
‘(Lit.) *The teacher recommended those us students.’				
(after Furuya 2008: 153, (13))

b.

**Table:** 

Papuan Malay							
*de*	*blang,*	*a,*	*[om*	***ko***	***ini*** *]*	*tra*	*liat…*
3sg	say	ah!	uncle	2sg	dem.prox	neg	see
‘he said: “Ah, you uncle here didn’t see…” ’							
(after Kluge 2017: 353, (66))

c.

**Table:** 

Amele					
*[Dana*	***i/eu***	***age*** *]*	*age*	*Hilu*	*dec.*
man	dem.prox/dem.dist	3pl	3pl		from
‘These/those men are from Hilu.’					
(after Roberts 1987: 217, (315))

d.

**Table:** 

Hausa			
*[* ***shī***	***wannàn***	*mùtumì-* ***n*** *]*	*kùwa…*
3sg	dem.prox	man-def	moreover
‘and (he) this man moreover…’			
(after Jaggar 2001: 331)

A last systematic deviation from the matching directionality of demonstratives and pers_n_ I want to address concerns the tendency for prenominal demonstratives among languages with postnominal BPCs (Section 4.2). Since those languages have postpositions and presumably a final pers_n_ head, the FOFC-based reasoning from Section 5.1 suggests that the prenominal demonstratives should occupy phrasal positions, e.g. the specifier of the pers_n_-head (48).

(48)

**Table:** 

[_PERSnP_ demP [_PERS__n__’_ [_NP_ …] pers_n_]]

This can capture the inapplicability of Hypothesis 2 in most BPC languages, since clitic pers_n_ markers and demonstratives are indeed not co-categorial. Correspondingly, PPDC-like co-occurrences of demonstratives and clitic pers_n_marking are expected and indeed attested for six of these 13 languages (Supplementary Material S3, Section 2.2, Table 7). Another type of expression may, however, show parallels to demonstratives after all. Several languages permit additional pers_n_-related expressions to co-occur in their BPCs (see Supplementary Material S3, Sections 1 and 2.2, Table 7). These may be simply pronouns (49) or, e.g. in Khoekhoe, article-like elements expressing a subset of person features (50), cf. (Haacke 1976, 1977, 2013).29Thanks to Watson Williams for help with Mi’kmaq glossing and to Menán du Plessis for Khoekhoe glossing. Khoekhoe glosses for pers_n_ expressions are my interpretation of Haacke (1977). These prenominal pers_n_-related markers seem to occupy the same position as the prenominal demonstratives; see the Khoekhoe demonstrative in (50b), suggesting co-categoriality for demonstratives with these expressions.30For Khoekhoe, Böhm (1985: 134f.) explicitly suggests that “[d]ie verschiedenen Pronominalstämme sind wahrscheinlich deiktischer Natur” (the different pronominal stems are probably deictic in nature). Assuming an analysis like (48), these patterns are also compatible with Jenks and Konate’s (2022) SIC.

(49)

**Table:** 

Mi’kmaq	
***ninen***	*elnui-* ***yek***
we.excl	people-1pl.excl
‘we First Nation people’	
(after Pacifique et al. 1990: 188)

(50)

**Table:** 

Khoekhoe

a.

**Table:** 

*[* ***ti***	*kḫoe-* ***ta*** *]*	*ké*	*ko*	*ro*	ǁ*’a-*ǁ*na.*
art.sg.auth	person-1sg	top?	recpst	prog	baptise
‘I for my part used to baptise.’					
(after Böhm 1985: 133, (26))

b.

**Table:** 

***nē***	*khoe-* ***gu***
dem.prox	person-3pl.m
‘these men’	
(after Haacke 1977: 54)

The analysis does not directly carry over to the four BPC languages with postnominal demonstratives: Basque, Windesi Wamesa, Menya and Moskona. Basque demonstratives occupy the same phrase-final position as – and are in complementary distribution with – the definite article, including the pers_n_/proximate plural article *-ok* (Section 3), so this seems to be an instance of co-categoriality properly in line with Hypothesis 2. Initial pronouns are obligatory at least in western varieties of Basque (Xabier Artiagoitia, pers. comm.) in examples like (51). The suffixal markers *-au* and *-ori* are equivalent to the close (dem.1) and medium (dem.2) distance demonstratives *hau* and *hori*. Artiagoitia (2012: 67) suggests that the initial pronouns occupy the specifier of the projection headed by the final demonstrative/pers_n_ marking, which parallels the structure in (46b), putting these data in line with the SIC.

(51)

**Table:** 

Basque

a.

**Table:** 

***ni***	*gizajo-* ***au***
I	poor-proxart
‘poor me’

b.

**Table:** 

***zu***	*txotxolo-* ***ori***
you	fool-proxart
‘you fool’	
(Artiagoitia 2012: 66, (100))

In the remaining three languages, demonstratives clearly realise distinct positions from pers_n_, potentially challenging the SIC. Windesi Wamesa demonstratives precede the determiner (Gasser 2014: 250), while the pers_n_ markers follow it (52). This implies distinct positions for both as sketched in (53). However, since they seem not to co-occur (Emily Gasser, pers. comm.), the pattern may be compatible with the SIC after all.

(52)

**Table:** 

Windesi Wamesa
*sinitu=pa-* ***tata***
person=det-1pl.incl
‘we people’
(Gasser 2014: 144, (3.46))

(53)

**Table:** 

N dem=det=pers_n_

In Menya, PPDCs are attested (54). While the prenominal pronoun probably occupies the specifier position of the final pers_n_ marker, the demonstrative and pers_n_ marking are structurally distinct, albeit part of a clitic cluster. Hence, co-categoriality is unlikely and issues remain for Jenks and Konate’s (2022) approach.

(54)

**Table:** 

Menya						
*[* ***Ne***	*ämaqä*	*qokä*	***i*** *=qu=* ***ne*** *]*	*yiämisaŋä*	*huiyi=nä*	*qw*
1pl	person	man	dem=m=1pl	food	other=foc	cert
*ä-n-k-qäqu=i.*						
ass-eat-pst/pfv-1pl/dso=ind						
‘Then we men ate some other food.’						
(Whitehead 2006: 30, (59))						

Moskona raises similar issues because it has immediately prenominal clitic pers_n_ marking and *x*nP-final demonstratives, which can co-occur (55a). The clitic pers_n _marking can also be preceded by a full pronoun (55b). While I found no examples containing all three deictic expressions simultaneously, three distinct loci are involved, raising the same issues for the SIC as the Hausa example (47d) above.

(55)

**Table:** 

Moskona

a.

**Table:** 

*[* ***I*** *-osnok*	*Mod*	*Ari*	***no-ma-i*** *]*	*erá*	*i-eg*	*mar…*
3pl-person	house	Sunday	dem-dist-giv	thm	3pl-hear	thing
‘The church people heard the thing…’						
(after Gravelle 2010: 224, (46))

b.

**Table:** 

*[* ***Eri***	***i*** *-ejen(a)]*	*i-odog*	*jig…*
they.pl	3pl-woman	3pl-pregnant	loc
‘(If/when) they the women are pregnant…’			
(after Gravelle 2010: 91, (38))

While the assumption that demonstratives and pers_n_ form one distributional category seems justified for a substantial number of languages, this section has addressed a range of apparent or real counterexamples. Some directionality mismatches can be explained by the occurrence of pers_n_ in marked demonstrative positions. The discussion of PPDCs has shown that matching directionality is not a sufficient condition for co-categoriality and that a number of languages seem to indeed encode pers_n_ separately from demonstrativity. Jenks and Konate (2022) propose that even such distinct realisations can be structurally packaged in specifier-head configurations to align with the SIC. This constraint is conceptually appealing and the analysis is probably applicable to a range of languages, but several datapoints are not obviously compatible with the required configurations. I therefore still consider analyses with distinct projections for pers_n_ and demonstratives to be a viable and empirically necessary option (Giorgi and Pianesi 1997; Hsu and Syed 2020; Höhn 2017).

## 6 Conclusions

This paper has investigated word order variation in the expression of pers_n_ in a sample of 113 languages. Two morphological types of pers_n_ marking were observed: adnominal pronoun constructions (APCs) and bound person constructions (BPCs). While most languages opt for one of these strategies, they turn out not to be mutually exclusive in principle. Table 5 repeats the observable interactions between the directionality of pers_n_ marking and characteristic indicators of head-directionality (adpositions, dependent genitives, verb-object order) and the directionality of demonstrative modifiers. Statistical testing of pre- and postnominal APCs data was coherent with Hypothesis 1 that pers_n_ tends to behave like a syntactic head with a tendency for head-initial characteristics in languages with prenominal APCs and head-final characteristics in languages with postnominal APC. In line with the pronominal determiner analysis, the relative directionality of APCs matches the relative directionality of articles in languages where the two are in complementary distribution (Hypothesis 1a). Moreover, demonstratives and adnominal pronouns tend to match in directionality relative to the lexical noun, consistent with the idea that they often form one distributional category (Hypothesis 2).

**Table 5:** Tentative word order tendencies for different types of pers_n_ marking.

	APC_pre_	APC_post_	APC_ambi_	BPC_post_
Genitives	Not clear	GenN	(GenN)	GenN
Adpositions	Prepositions	Postpositions	(Postpositions)	Postpositions
Verb-object order	VO	OV	OV	OV
Demonstratives	(DemN)	NDem	(NDem)	DemN

Deviations from Hypotheses 1 and 2 support the notion sketched in Section 2.2 that pers_n_ being a head and its categorial identity with demonstratives are not universals, but points of variation. While the data are consistent with pers_n_ being widely encoded as head, a subclass of languages with prenominal APCs and indications of head-finality may encode pers_n_ in specifiers or as adjuncts (Section 5.1). Similarly, pers_n_ and demonstratives forming a distributional class appears to be the preferred strategy for languages, but there are systematic exceptions especially in languages with pers_n_/pronoun-demonstrative constructions (PPDCs) suggesting that pers_n_ and demonstrativity can also be encoded separately (Section 5.3).

This research only offers a first look at the empirical landscape of pers_n_. Future research into a wider range of languages should help to verify, extend and, if necessary, amend the picture presented here. The syntactic analyses mentioned especially in Section 5 are only sketches for reasons of space, so detailed investigation of pers_n_ in specific languages is desirable to verify the adequacy of the suggestions made here. This particularly also concerns PPDCs, which offer important data for investigating variation in the bundling of syntactic features onto syntactic heads (Hsu 2021; Hsu and Syed 2020) and may provide a basis for connecting research on pers_n_ with existing proposals regarding Greenberg’s Universal 20 (Abels and Neeleman 2012; Cinque 2005).

## Supplementary Material

– S1.csv: table with annotated language data
– S2.R: script used for statistical analysis and map visualisation
– S3.pdf: document containing
  – discussion of languages with postnominal and ambidirectional APCs; BPCs
  – supporting tables for Sections 5.1 and 5.3
  – model selection for statistical analysis (Section 4.3)
  – list of surveyed languages with bibliographic references for information on pers_n_
  – description of columns in S1
  – glossed pers_n_ examples for all languages in sample

## Supplementary Material

Supplementary Material

## Abbreviations

abs: absolutive
acc: accusative
AIC: Akike information criterion
all: allative
APC: adnominal pronoun construction
art: article
ass: assertive
auth: author
aux: auxiliary
BPC: bound person construction
cert: certainty of assertion
com: comitative
comp: complementizer
dat: dative
def: definite
dem: demonstrative
det: determiner
dist: distal
dso: dissociative
dur: durative
emph: emphatic
erg: ergative
excl: exclusive
f: feminine
foc: focus
FOFC: Final-over-Final Condition
gen: genitive
giv: given/known information
group: referent forms a group
hum: human
imp: imperative
incl: inclusive
ind: indicative
ipfv: imperfective
lig: ligature
lnk: adnominal linker
loc: locative
m: masculine
n: neuter
nact: non-actual
neg: negative
nf: non-finite
nmlz: nominalizer
nom: nominative
obj: object
pers_n_: adnominal person
pfv: perfective
pl: plural
png: person-number-gender
poss: possessive
PPDC: pers_n_/pronoun-demonstrative construction
pref: prefix
prog: progressive
prox: proximal
prxart: proximate article
pst: past
recpst: recent past
redup: reduplication
sg: singular
SIC: single index constraint
thm: thematic marker
TNG: Trans-New Guinea
todpst: today’s past
top: topic
xnp: extended nominal projection.
