# Supplementary material for: A word order typology of adnominal person
Source: Linguist Typol. 2024 Nov 4;29(1):35–80. doi: 10.1515/lingty-2023-0080 (PMC12288869; doi:10.1515/lingty-2023-0080)
Supplement: Supplementary file 1 — Supplementary Material [file j_lingty-2023-0080_suppl_001.zip › supplementary files/S3.pdf]

...

Georg F.K. Höhn

A word order typology of adnominal person

Supplementary documentation

1 Notes on postnominal and ambidirectional adnominal pronoun constructions (APCs) and bound person constructions (BPCs)

Table 1 lists languages with postnominal APCs with their word order properties. Several examples were already presented in Section 2.3 of the main paper.

Tab. 1: Word order properties of languages with postnominal pronouns

Language	Classification	Adpos.	Genit.	WO	Demonstr.
Warlpiri	Western Pama-Nyungan (PN)	post	GenN	NC	NDem
Lavukaleve	Lavukaleve	post	GenN	OV	NDem
Maybrat	Maybrat	pre	NoDom	VO	NDem
Savosavo	Savosavo	post	GenN	OV	DemN
Yagaria	Trans-New Guinea (TNG), Siane-Yagaria	post	GenN	OV	DemN
Amele	TNG, Mabuso	post	GenN	OV	NDem
Adang	TNG, Alor-Pantar	post	GenN	OV	NDem
Kaera	TNG, Alor-Pantar	post	GenN	OV	NDem
Kamang	TNG, Alor-Pantar	NoDom	GenN	OV	NDem
Sawila	TNG, Alor-Pantar	NoAdpos	GenN	OV	NDem
Wersing	TNG, Alor-Pantar	NoAdpos	GenN	OV	NDem
Western Pantar	TNG, Alor-Pantar	post	GenN	OV	NDem
Papuan Malay	Austron., Malayo-Sumbawan	pre	GenN	VO	NDem
Kalaallisut	Eskimo	post	GenN	OV	mixed
East Geshiza	Sino-Tibetan, Burmo-Qiangic	post	GenN	OV	DemN

Georg F.K. Höhn, Georg-August-University Göttingen

Fortescue (1984: 257) presents Kalaallisut as having prenominal APCs based on (1b), which, however, involves a participial construction and is therefore presumably not ad-*nominal*. I classify the language as having postnominal APC on the basis of examples like (1a) with a plain nominal.

(1) a. *kalaalli-t* ***uagut*** *[Kalaallisut]*
Greenlander-ABS.PL we
'we Greenlanders'
(after Fortescue 1984: 110; gloss extrapolated)

b. ***uagut*** *kalaali-u-sugut*
we Greenlander-be-1PL.PTCP
'we Greenlanders'
(after Fortescue 1984: 257)

A postnominal APCs from East Geshiza is illustrated in (2). Apart from several postnominal APC examples the grammar contains a single example of a prenominal APC (Honkasalo 2019: 388, (5.74)), which incidentally also contains a numeral modifier. I still classify the language as having postnominal APCs, since this is clearly the default option (Sami Honkasalo, p.c.) and the significance of the exceptional datapoint is currently unclear.

(2) *[bæ* ***ŋæ***=ɲə=*tʰə]* *'mbəzli'* *d-ə-joŋ.* *[East Geshiza]*
Tibetan 1=PL=TOP ritual.tripod PREF-NACT-say.1
'We Tibetans call it *mbəzli* (ritual tripod).'
(Honkasalo 2019: 400, 5.115)

The status of ambidirectional APCs is not entirely clear due to limited available information on the relevant constructions, but the seven languages in Table 2 appear to allow both pre- and postnominal pronouns, with the available description or data providing no clear indication whether one of them behaves as a default.

Tab. 2: Languages with ambidirectional APCs

Language	Genus	Adpos.	Genit.	WO	Demonstr.
Pitjantjatjara	Western PN	post	GenN	OV	NDem
Guugu Yimidhirr	Northern PN	unclear	NoDom	OV	DemN
Kuku-Yalanji	Northern PN	pre	NoDom	OV	mixed
Imonda	Border	post	GenN	OV	mixed
Usan	North Adelbert	post	GenN	OV	NDem
Kobon	TNG, Kalam-Kobon	post	GenN	OV	NDem
Katu	Katuic	pre	NGen	unclear	NDem

Katu is the only language in the sample with evidence of pre- and postnominal pronouns occurring simultaneously, cf. (3c).[1] Unfortunately, Costello (1969) does not discuss what determines the choice between the options in (3).

(3) a. *manuih* ***yi*** *[Katu]*
people we

b. ***yi*** *manuih*
we people
'we people'

c. ***yi*** *adi* *anó* ***yi***
we older.brother younger.brother we
'we older and younger brothers'
(Costello 1969: 28, (35–37))

For some other languages, information structure might influence the choice between pre- or postnominal APCs. For Kuku Yalanji, Patz (2002: 202) suggests that prenominal pronouns "can be assumed to have anaphoric or definite reference", while postnominal pronouns "normally [establish] a new reference". Referring to personal communication from Paul Eckhert, Bowe (1990: 34) suggests that prenominal (as opposed to the unmarked postnominal) demonstratives in Pitjantjatjara "seem to imply contrast". Considering the translation of (4b), prenominal *paluṟu* appears to have the same effect. While one might speculate that postnominal APCs also represent the unmarked order, in the absence of explicit confirmation I classify Pitjantjatjara as having ambidirectional APCs for now.

(4) a. *[Minyma* ***paluṟu****]* *ngayu-nya* *nya-ngu* *[Pitjantjatjara]*
woman 3SG.NOM 1SG.ACC see-PST
'The woman saw me.'
(Bowe 1990: 31, (110))

b. *[****Paluṟu*** *wati* *nyara* *waṟa-ngku]* *mutaka* *palya-nu*
3SG.NOM man DEM.3 tall-ERG car fix-PST
'The tall man over there (in contrast to the other one) fixed the car.'
(after Bowe 1990: 34, (114))

Finally, Reesink (1987: 53f., 167) describes *postnominal* APCs in Usan (5a) as indicative of contrastive topics, topic shift or emphasis on topics. However, prenominal APCs are not specifically addressed in the grammar at all. Example (5b) occurs as part of a discussion on sentence connectors and the second sentence picks up the object of

1 For other instances of double marking of adnominal person (PERS$_{\text{N}}$) see Table 3 below.

the first clause as APC subject with a prenominal APC, suggesting compatibility with topic shift, too. The significance of APC order in Usan thus remains unclear.

(5) a. *eâb igim-ine ne [tain* ***wo****] yâ-nâmb wogub…* *[Usan]*
cry.SS be-1SG.DS and father he me-hit.SS cease.SS
'I was crying and my father he hit me and then…'
(after Reesink 1987: 167, (99))

b. *… in bo* ***[an*** *wau moi] qomon gâs ende ig-oun. Ne*
we again you.PL child unmarried custom like thus be-1PL.PRS and
[an *munon moi e]…*
you.PL man unmarried DEM.PROX
'…we in turn live like the customs of you young men. And you young men here…'
(initial part of Reesink 1987: 190f., (41))

Morphologically bound PERS$_{N}$ marking is attested in the languages in Table 3. Note that Bilua (6) and Windesi Wamesa (7) are the only VO languages with postnominal BPCs. VO order in Bilua probably results from contact with Austronesian languages (Terrill 2011: 323), see Figure 3 in the main paper. Being Austronesian, VO order is unsurprising for Windesi Wamesa, but it is the only Austronesian language in the sample with BPCs. It seems no coincidence that it is spoken in the same area of intense contact between Austronesian and non-Austronesian languages as Papuan Malay, the only Austronesian language in the sample with postnominal APCs (Table 1) and itself an important contact language for Windesi Wamesa (Gasser 2014: 68f.; Kluge 2017: 27–33), see Figure 2 in the main paper.

(6) *enge=ko visi=****nga*** *[Bilua]*
1PL.EXCL=3SG.F younger.sibling=2SG
'you who are our younger sister'
(Obata 2003: 103, (7.116))

(7) *sinitu=pa-****tata*** *[Windesi Wamesa]*
person=DET-1PL.INCL
'we people'
(Gasser 2014: 144, (3.46))

Six languages, marked by [c] in Table 3, also seem to permit an additional prenominal pronoun(-like) PERS$_{N}$-marking in BPCs (for further discussion see Sections 5.1 and 5.3 in the main paper). For the three BPC languages marked with [a] and [b] in Table 3 there is some evidence for independent APCs as well.

PERS$_{N}$ in Moskona is marked by prefixes on "[g]eneric nouns which denote humans" (Gravelle 2010: 219), see (8a). Adjectival (and numeral) modifiers also seem to show

Tab. 3: Languages with BPCs
[a]: also has prenominal APCs, [b]: also has postnominal APCs, [c] allows prenominal pronouns in BPCs.

Language	Classification	Adpos.	Genit.	WO	Demonstr.
prenominal BPCs					
Classical Nahuatl	Aztecan	NoAdpos	unclear	NC	mixed
Moskona[ac]	East Bird's Head	pre	GenN	VO	NDem
postnominal BPCs					
Menya[c]	Angan, Nuclear Angan	post	GenN	OV	NDem
Bilua[ac]	Central Solomons, Bilua	post	GenN	VO	DemN
Alamblak	Sepik Hill	post	GenN	OV	DemN
Fore	TNG, Fore-Gimi	post	GenN	OV	DemN
Hua	TNG, Siane-Yagaria	post	GenN	OV	DemN
Yagaria[b]	TNG, Siane-Yagaria	post	GenN	OV	DemN
Windesi Wamesa	Austronesian, Oceanic	pre	GenN	VO	NDem
Mi'kmaq[c]	Algonquian	NoDom	GenN	NC	DemN
Khoekhoe (Nama)[c]	Khoe-Kwadi	post	GenN	OV	DemN
Khwe/Kxoe[c]	Khoe-Kwadi	post	GenN	OV	DemN
Basque[c]	Basque	post	GenN	OV	NDem

person-number agreement (Gravelle 2010: 127), see (8b). Additionally, Gravelle (2010: 224) shows examples containing only a prenominal APC without the prenominal PERS$_N$ marker (8c). They generally contain proper names, so maybe those are incompatible with Moskona's PERS$_N$ prefixes.

(8) a. ***mi***-*osnok* *mi-en-ah-miy,* *mi-en-ot* *jig* *miyes* *[Moskona]*
1PL-person 1PL-DUR-lie-water 1PL-DUR-stand LOC clothes

mi-er *tofi.*
1PL-wear hat

'we people bathe, wear clothes, wear hats…' (stand in clothes = wear clothes)
(after Gravelle 2010: 344, (2))

b. ***i***-*osnok* ***i***-*ofogo*
3PL-person 3PL-evil

'evil people'
(Gravelle 2010: 194, (26a))

c. ***eri*** *Mosmir*
they Maybrat

'Maybrat people/tribe'
(Gravelle 2010: 224, (43b))

Independently of the postnominal BPC mentioned above (6), Bilua also seems to allow prenominal APCs (9). The ligature marker "occurs only between morphemes which belong to the same phrase" (Obata 2003: 79), suggesting that (9) does not involve apposition of two distinct noun phrases.

(9) ***enge***=*a* *saidi* *[Bilua]*
1PL.EXCL=LIG family
'we, family'
(Obata 2003: 79, (7.10))

Finally, Yagaria has postnominal BPCs (10a), but also uses postnominal pronouns "in focused phrases [. . .], especially in transitive clauses where the marking of the subject is obligatory" (Renck 1975: 17). The construction is also available with focused objects, see (10b), making an analysis of the postnominal pronoun as a resumptive less likely, since the noun *ve* is not left-peripheral here.

(10) a. *[Ovu-**da**]* *ma-lo'* *bei-d-u-e* *[Yagaria]*
Ovi-I this-LOC live-PST-1.SG-IND
'I, Ovu, am here.'

b. *dagaea* *[ve* ***agaea**]* *∅-begi-d-u-e*
I man he him-hit-PST-1SG-IND
'I hit the man.'
(Renck 1975: 18f.)

Closely related Fore might also involve postnominal APCs, although Scott (1978: 100) notes the possibility of an intonational break before the "pronominal copy" indicated by the comma in (11). Since a resumptive analysis cannot be excluded at least for this example, I treat Fore as only having postnominal BPCs for now, cf. (5) in the main paper.[2]

(11) *[teméni'-N* *a-pa:'],* *[**áe'**]* *kana-y-e* *[Fore]*
Temeni-OBL his-father he come-he-IND
'Temeni's father is coming.'
(after Scott 1978: 100, (163a))

2 Classifying Fore as language with postnominal APCs would only strengthen the tendencies observed in the next section.

2 Supporting tables

2.1 For Section 5.1

Tab. 4: Languages with prenominal APCs and at least two indicators of head-finality

Language	Classification	Genit.	Adpos.	WO
Bilua	Bilua	GenN	post	VO
Hungarian	Uralic, Ugric	GenN	post	VO
Finnish	Uralic, Finnic	GenN	post	VO
Japanese	Japanese	GenN	post	OV
Korean	Korean	GenN	post	OV
Evenki	Tungusic	GenN	post	OV
Turkish	Turkic	GenN	post	OV
Momu	Baibai-Fas	GenN	post	OV
Manambu	Sepik, Ndu	GenN	post	OV
Awtuw	Sepik, Ram	GenN	post	OV
Chitimacha	Chitimacha	GenN	post	OV
Kannada	Dravidian	GenN	post	OV
Malayalam	Dravidian	GenN	post	OV
Tamil	Dravidian	GenN	post	OV
Kashmiri	Indo-European (IE), Indic	GenN	post	OV
Marathi	IE, Indic	GenN	post	OV
Punjabi	IE, Indic	GenN	post	OV
Supyire	Niger-Congo, Senufo	GenN	post	OV
Lezgian	Lezgic	GenN	post	OV
Abkhaz	Northwest Caucasian	GenN	post	OV
Kambaata	Afroasiatic, East Cushitic	GenN	NoAdpos	OV
Diyari	Central PN	GenN	unclear	OV

2.2 For Section 5.3

Tab. 5: Languages with mismatch in directionality of APCs and demonstrative modifiers. In column personal pronoun-demonstrative construction (PPDC), (✓) indicates that the attested PPDC examples involve no overt noun.

Language	Classification	Alt. DEM position	PPDC
prenominal APCs and NDem			
Momu	Baibai-Fas		✓Honeyman 2016: 559
Moskona	East Bird's Head		✓Gravelle 2010: 187
Sougb	East Bird's Head		✓Reesink 2002: 271
Hatam	Hatim-Mansim		✓Reesink 1999: 41, 195
Urim	Urim		✓Hemmilä &Luoma 1987: 140
Mupun	Afroasiatic		
Gorwaa	Afroasiatic		✓Harvey 2018: 116
Cair. Eg. Arabic	Afroasiatic	(✓) Doss 1979: 350f.[3]	
Indonesian	Austronesian		(✓) Sneddon 1996: 169
Loniu	Austronesian		✓Hamel 1994: 100
Tuvaluan	Austronesian		(✓) Besnier 2000: 409
Kwaio	Austronesian		
Arop-Lokep	Austronesian		✓D'Jernes 2002: 255
Cheke Holo	Austronesian	✓Boswell 2018: 169	✓Boswell 2018: 165
Hoava	Austronesian		✓Davis 2003: 48
Kokota	Austronesian		✓Palmer 2008: 116
Wari'	Chapacura-Wanham		
Ndyuka	Creoles and Pidgins		(✓) Huttar &Huttar 1994: 203, 329
Welsh	IE		
Luganda	Niger-Congo	✓Ashton et al. 1954: 41	
Nkore-Kiga	Niger-Congo	✓Tayebwa 2014: 10	
Nzadi	Niger-Congo	✓Crane et al. 2011: 100	
Swahili	Niger-Congo	✓Mpiranya 2015: 35	
Babungo	Niger-Congo	✓Schaub 1985: 73	
Koromfe	Niger-Congo		✓John Rennison, p.c.
Lakkia	Kadai		
postnominal APCs and DemN			
Savosavo	Savosavo		(✓) Wegener 2012: 86
Yagaria	TNG		
East Geshiza	Sino-Tibetan	✓Honkasalo 2019: 40	✓(2), Honkasalo 2019: 301f., 400

Tab. 6: PPDCs in languages with APCs with matching demonstrative directionality. (✓) indicates that attested PPDCs contained no overt noun.

Language	Classification	DEM order	PPDC
prenominal APCs			
Japanese	Japanese	DemN	(✓) Coulmas 1982: 214; Furuya 2008: 153; Noguchi 1997: 777
Korean	Korean	DemN	(✓) Sohn 1994: 281
Diyari	Central PN	DemN	(✓) Austin 1981: 61, (36)
Kayardild	Tangkic	DemN	✓Evans 1995: 251, (6-37)
Manambu	Sepik, Ndu	DemN	✓Aikhenvald 2008: 198
Pomak	IE	DemN	(✓) Papadimitriou 2008: 581
Mandarin	Sino-Tibetan	DemN	✓Huang et al. 2009: 298f.
Hausa	Afroasiatic	mixed	✓Jaggar 2001: 331; Newman 2000: 155, 371
Maori	Austronesian	mixed	(✓) Bauer 1997: 263f.
Malagasy	Austronesian	both	✓Paul &Travis 2019: 422
postnominal APCs			
Lavukaleve	Lavukaleve	NDem	(✓) Terrill 2003: 181, (220-1)
Amele	TNG	NDem	✓(16) in main text, Roberts 1987: 210, 218
Kaera	TNG	NDem	✓Klamer 2014: 129, (99)
Papuan Malay	Austronesian	NDem	✓Kluge 2017: 353, (66/67)
ambidirectional APCs			
Kuku-Yalanji	Northern PN	mixed	✓Patz 2002: 204, (625)
Guugu Yimidhirr	Northern PN	DemN	✓Haviland 1979: 73, (107); 157, (423); 160
Pitjantjatjara	Western PN	NDem	✓Bowe 1990: 48–51
Usan	TNG	NDem	✓(5b), Reesink 1987: 190, (141)

3 Following Doss (1979: 351) DemN orders in Colloquial Egyptian Arabic are highly restricted and “constitute residuals from a previous stage during which the variation of word-order was a freer one.”

Tab. 7: Languages with BPC allowing additional adnominal PERS$_N$-marking and/or PPDCs. For (✓) the attested PPDCs involve no overt noun.

Language	Classification	DEM order	Pron. in BPC	PPDC
Postnominal BPC				
Khoekhoe	Khoe-Kwadi	DemN	pre	✓Haacke 1977: 54
Khwe/Kxoe	Khoe-Kwadi	DemN	pre	✓Kilian-Hatz 2008: 41, (2/3); 49, (25)
Menya	Nuclear Angan	NDem	pre	✓Whitehead 2006: 30, (58/59)
Alamblak	Sepik	DemN		✓Bruce 1984: 90, (149a)
Bilua	Bilua	DemN	pre	✓Obata 2003: 289, (8)
Mi'kmaq	Algonquian	DemN	pre	
Basque	Basque	NDem	pre	
Prenominal BPC				
Moskona	East Bird's Head	NDem	pre	✓Gravelle 2010: 187, 224

3 Model selection for random intercept structure

As discussed in Section 4.3 of the main text, the generalised linear mixed-effect model to be developed involves APC directionality as dependent variable and the two fixed effects HEADFIN (for head-directionality) and DEMFIN (for noun-demonstrative order), see (12). This additional section discusses the method used for determining the random effect structure.

(12) APCDIR $\sim$ HEADFIN + DEMFIN + *random effects*

Two types of random effects to consider for inclusion into the target model are phylogenetic relationships between the languages in the sample and their geographical clustering/distance. To asses the impact of phylogenetic distance, two levels of detail were considered, language FAMILY (f) and GENUS (g) following, e.g. Dryer (1989) and the notation in WALS (Dryer &Haspelmath 2013). In addition to either of the two phylogenetic descriptors in isolation as random intercepts, models were also fitted with GENUS nested under FAMILY in order to capture the phylogenetic structure. In order to assess potential areal effects and language contact, I calculated the distances between languages based on their coordinates (spherical distances, calculated with the function function `distm`, `geosphere` package Hijmans 2022). The distance matrix between the locations of the sample languages was reduced to one and two dimensions with multidimensional scaling (`cmdscale` function). The estimates of these two dimensions for each language were used as random intercepts dist.1 and dist.2 in (42). The estimates of the scaling to two dimensions (2D) were coded using two random intercepts MDS1 and MDS2. An alternative with reduction to one dimension (1D) was also considered with the estimate coded by the random intercept MDS.SINGLE.

The various combinations of these random intercept structures are listed in Table 8, with the dependent variable and fixed effects from (12) replaced by … for brevity. Models marked with * did not converge and models marked with ! had a singular fit, indicating overfitting.

In order to identify a random effect structure that appropriately balances modelling detail and overfitting, the converging models are compared in an ANOVA in Table 9.

The model comparison indicates that model `mdir.g` with only GENUS as random intercept has the lowest Akike information criterion (AIC), so the analysis in the main text adopts the model in (13).

Tab. 8: Random effect structures for (12)

	issues	model		random effects
a.		mdir.2Dfg	= ... +	(1 | MDS1) + (1 | MDS2) + (1 | FAMILY/GENUS)
b.	*	mdir.2Df	= ... +	(1 | MDS1) + (1 | MDS2) + (1 | FAMILY)
c.		mdir.2Dg	= ... +	(1 | MDS1) + (1 | MDS2) + (1 | GENUS)
d.	!	mdir.2D	= ... +	(1 | MDS1) + (1 | MDS2)
e.	!	mdir.1Dfg	= ... +	(1 | MDS.SINGLE) + (1 | FAMILY/GENUS)
f.		mdir.1Df	= ... +	(1 | MDS.SINGLE) + (1 | FAMILY)
g.		mdir.1Dg	= ... +	(1 | MDS.SINGLE) + (1 | GENUS)
h.	!	mdir.1D	= ... +	(1 | MDS.SINGLE)
i.		mdir.fg	= ... +	(1 | FAMILY/GENUS)
j.		mdir.f	= ... +	(1 | FAMILY)
k.		mdir.g	= ... +	(1 | GENUS)

Tab. 9: Model selection among converging models from Table 8

		npar	AIC	BIC	logLik	deviance	χ^2	Df	Pr(>Chisq)
	mdir.1D	4	47.414	57.671	-19.707	39.41			
	mdir.f	4	29.418	39.675	-10.709	21.42	18.00	0	
☞	**mdir.g**	4	**29.398**	39.655	-10.699	21.40	0.02	0	
	mdir.2D	5	49.414	62.236	-19.707	39.41	0.00	1	1.00
	mdir.1Dg	5	31.398	44.220	-10.699	21.40	18.02	0	
	mdir.1Df	5	31.418	44.240	-10.709	21.42	0.00	0	
	mdir.fg	5	31.396	44.218	-10.698	21.40	0.02	0	
	mdir.2Dg	6	33.398	48.784	-10.699	21.40	0.00	1	1.00
	mdir.1Dfg	6	33.418	48.804	-10.709	21.42	0.00	0	
	mdir.2Dfg	7	35.398	53.348	-10.699	21.40	0.02	1	0.89

(13) APCDIR $\sim$ HEADFIN + DEMFIN + (1 | GENUS)

4 List of surveyed languages

Tab. 10: List of sampled languages
Grammars based on Comrie &Smith's (1977) questionnaire are marked (Q). Genus information is based on the corresponding classification in WALS (Dryer &Haspelmath 2013). For languages without WALS entry (marked by *) genus is extrapolated from glottolog (Hammarström et al. 2021) or the respective grammars.

Glottocode	Language	Genus	Main source(s)
Afroasiatic languages (7 languages/4 genera)			
haus1257	Hausa	West Chadic	Newman 2000: 63, 155, 370f.; Jaggar 2001: 330f.
mwag1236	Mupun	West Chadic	Frajzyngier 1993: 172
goro1270	Gorwaa	Southern Cushitic	Harvey 2018: 163
kamb1316	Kambaata	East Cushitic*	Treis 2008: 335
egyp1253	Cairene Egypt. Arabic	Semitic	(Q) Gary &Gamal-Eldin 1982: 78, 80
gulf1241	Gulf Arabic	Semitic	(Q) Holes 1990: 162, 165
malt1254	Maltese	Semitic	(Q) Borg &Azzopardi-Alexander 1997: 187f., 202
Australian languages (7 languages/5 genera)			
mang1381	Mangarrayi	Mangarrayi	(Q) Merlan 1989: 103; 203
pirl1241	Diyari	Central PN	Austin 1981: 97f., (Austin 2013: 102f.)
warl1254	Warlpiri	Western PN	Reece 1970: 70; Hale 1973: 316f.
pitj1243	Pitjantjatjara	Western PN	Bowe 1990: 49–51
gugu1255	Guugu Yimidhirr	Northern PN	Haviland 1979: 104, 156f.
kuku1273	Kuku Yalanji	Northern PN	Patz 2002: 120f., 202f.
kaya1319	Kayardild	Tangkic	Evans 1995: 239; 251; Round 2013: 141
Austronesian languages (12 languages/3 genera)			
mala1537	Malagasy	Barito	Paul &Travis 2019
stin1234	Indonesian	Malayo-Sumbawan	Sneddon 1996: 170
papu1250	Papuan Malay	Malayo-Sumbawan	Kluge 2017: ch. 6.2
loni1238	Loniu	Oceanic	Hamel 1994: 90
wame1241	Windesi Wamesa	Oceanic*	Gasser 2014: 144
maor1246	Maori	Oceanic	(Q) Bauer 1993: 368, 373; Bauer 1997: 262f.
tuva1244	Tuvaluan	Oceanic	(Q) Besnier 2000: 392f.
kwai1243	Kwaio	Oceanic	Keesing 1985: 104
arop1243	Arop-Lokep	Oceanic	D'Jernes 2002: 255
chek1238	Cheke Holo	Oceanic	Boswell 2018: 165

Tab. 10: (continued)

Glottocode	Language	Genus	Main source(s)
hoav1238	Hoava	Oceanic	Davis 2003: 47; Palmer 2017
koko1269	Kokota	Oceanic	Palmer 2008: 68, 95, 116, 119, 123, 131, 137, 163, 242, 300, 305, 327, 399, 414
		Dravidian languages (3 languages/1 genus)	
nucl1305	Kannada	Dravidian	(Q) Sridhar 1990: 205, 208f.
mala1464	Malayalam	Dravidian	(Q) Asher &Kumari 1997: 262f.
tami1289	Tamil	Dravidian	(Q) Asher 1985: 142, 146
		Indo-European languages (27 languages/7 genera)	
wels1247	Welsh	Celtic	David Willis (p.c.)
dani1285	Danish	Germanic	Johannessen 2008; Schröter 2021
icel1247	Icelandic	Germanic	Johannessen 2008; Sigurðsson &Wood 2020: 11f.
norw1258	Norwegian	Germanic	Julien 2005: 127, 129; Johannessen 2008
swed1254	Swedish	Germanic	Julien 2005: 128; Johannessen 2008
dutc1256	Dutch	Germanic	Corver 2008: 52
stan1293	English	Germanic	Delorme &Dougherty 1972; Keizer 2016; Pesetsky 1978; Postal 1969; Sommerstein 1972
stan1295	German	Germanic	Lawrenz 1993: ch. 6; Rauh 2003, 2004; Roehrs 2005
mode1248	Std. Mod. Greek	Greek	Choi 2014: chs. 1/2; Höhn 2016: sec. 5
apul1236	Calabrian Greek/Greko	Greek*	Höhn et al. 2017: 274
kash1277	Kashmiri	Indic	(Q) Wali &Koul 1997: 200
mara1378	Marathi	Indic	(Q) Pandharipande 1997: 386
panj1256	Punjabi	Indic	(Q) Bhatia 1993: 228
west2369	Persian	Iranian	(Q) Mahootian 1997: 209, 212
arom1237	Aromanian	Romance*	Höhn 2016: 546, 560
roma1327	Romanian	Romance	(Q) Mallinson 1986: 255, 258, Cornilescu &Nicolae 2014: 6, 10, 20f.
stan1289	Catalan	Romance	(Q) Hualde 1992: 287, 290; Höhn 2016: 560

Tab. 10: (continued)

Glottocode	Language	Genus	Main source(s)
gali1258	Galician	Romance	Álvarez et al. 1986: 152, 301; Höhn 2016: 560
port1283	(Eur.) Portuguese	Romance	Höhn 2016: 555, 560
stan1288	Spanish	Romance	de Bruyne 1995: 145; Choi 2014: 210f.; Höhn 2016: 560
ital1282	Italian	Romance	Cardinaletti 1994: 202f.; Höhn 2016: 559
nort2612	Northern Calabrese	Romance*	Höhn et al. 2016: 142
sout2616	Southern Calabrese	Romance*	Höhn et al. 2016: 142
russ1263	Russian	Slavic	Pesetsky 1978: 352
bulg1262	Bulgarian	Slavic	Höhn 2016: 560
poma1238	Pomak	Slavic*	Papadimitriou 2008: 582
poli1260	Polish	Slavic	Rutkowski 2002: 161
		Khoe-Kwadi languages (2 languages/1 genus)	
nama1264	Khoekhoe (Nama)	Khoe-Kwadi	Böhm 1985: 133–145; Haacke 2013, 1976, 1977; Himmelmann 1997: 215f.; Lyons 1999: 142f., 311; Maho 1998: 140; Rust 1965: 18
kxoe1243	Khwe (Kxoe)	Khoe-Khwadi	Kilian-Hatz 2008: 40–43, 79
		Niger-Congo languages (8 languages/4 genera)	
kiny1244	Kinyarwanda	Bantu	van der Wal 2022: 68f.
gand1255	Luganda	Bantu	Ashton et al. 1954: 102
nkor1241	Nkore-Kiga	Bantu	(Q) Taylor 1985: 131
nzad1234	Nzadi	Bantu*	Crane et al. 2011: 210, 279
swah1253	Swahili	Bantu	Vital Kazimoto (p.c.)
veng1238	Babungo	Wide Grassfields	(Q) Schaub 1985: 197f.
koro1298	Koromfe	Koromfe	(Q) Rennison 1997: 242, 250f.
supy1237	Supyire	Senufo	Carlson 1994: 207f.
		Papuan languages (26 languages/19 genera)	
meny1245	Menya	Nuclear Angan	Whitehead 2006: 40, 46, 56f.; Whitehead 2013: 9f., 18f.
fass1245	Momu	Baibai-Fas	Honeyman 2016: 169f., 242, 568
imon1245	Imonda	Border	Seiler 1985: 44, 61f.
bilu1245	Bilua	Bilua	Obata 2003: 47–49, 76, 79, 84f., 87–89, 92f.

Tab. 10: (continued)

Glottocode	Language	Genus	Main source(s)
lavu1241	Lavukaleve	Lavukaleve	Terrill 2003: 171–173
mosk1236	Moskona	East Bird's Head*	Gravelle 2010: 91, 222–224, 344
mani1235	Sougb	East Bird's Head	Reesink 2002: 200, 269f., 274
hata1243	Hatam	Hatim-Mansim	Reesink 1999: 195
maib1239	Maybrat	Maybrat	Dol 2007: 141, 158, 172, (281)
urim1252	Urim	Urim	Hemmilä &Luoma 1987: 123, 125
savo1255	Savosavo	Savosavo*	Wegener 2012: 147,155–159
mana1298	Manambu	Sepik, Ndu	Aikhenvald 2008: 197f., 508–513
awtu1239	Awtuw	Sepik, Ram	Feldman 1986: 120–124
alam1246	Alamblak	Sepik, Sepik Hill	Bruce 1984: 90–92, 96f.
fore1270	Fore	TNG, Fore-Gimi	Scott 1978: 79f., 100f.
huaa1250	Hua	TNG, Siane-Yagaria	Haiman 1980: 226–232, 239f.
yaga1260	Yagaria (*Move*)	TNG, Siane-Yagaria	Renck 1975: 17f., 166, 181
amel1241	Amele	TNG, Mabuso	(Q) Roberts 1987: 162, 201, 209f.
kobo1249	Kobon	TNG, Kalam-Kobon	(Q) Davies 1989: 107f., 157
usan1239	Usan	TNG, North Adelbert	Reesink 1987: 53f., 167, 190f., 353
adan1251	Adang	TNG, Alor-Pantar	Robinson &Haan 2014: 261
kaer1234	Kaera	TNG, Alor-Pantar*	Klamer 2014: 129
kama1365	Kamang	TNG, Alor-Pantar	Schapper 2014: 313f.
sawi1256	Sawila	TNG, Alor-Pantar*	Kratochvíl 2014: 391
wers1238	Wersing	TNG, Alor-Pantar	Schapper &Hendery 2014: 472
lamm1241	Western Pantar	TNG, Alor-Pantar*	Holton 2014: 53f.
		Sino-Tibetan (2 languages/2 genera)	
gesh1238	East Geshiza	Burmo-Qiangic*	Honkasalo 2019: 388, 400, 438, 480, 507, 646
mand1415	Mandarin	Chinese	Huang et al. 2009: 297–299; Bošković &Hsieh 2013: sec. 7.3
		Uralic (2 languages/2 genera)	
hung1274	Hungarian	Ugric	(Q) Kenesei et al. 1998: 269; Höhn 2016: 559
finn1318	Finnish	Finnic	(Q) Sulkala &Karjalainen 1992: 277
		Creoles (3 languages/1 "genus")	
ndyu1242	Ndyuka	Creoles	(Q) Huttar &Huttar 1994: 224, 460, 466f.
nige1257	Nigerian Pidgin	Creoles	(Q) Faraclas 1996: 178, 181

Tab. 10: (continued)

Glottocode	Language	Genus	Main source(s)
mala1533	Kristang	Creoles	Baxter 1988: 86
		Various (15 languages/15 genera)	
mikm1235	Mi'kmaq	Algonquian	Pacifique et al. 1990: 188
nucl1297	Katu	Katuic	Costello 1969: 28
wari1268	Wari'	Chapakuran	(Q) Everett &Kern 1997: 303, 310
chit1248	Chitimacha	Chitimacha	Swadesh 1967: 333
lezg1247	Lezgian	Lezgic	Haspelmath 1993: 259
abkh1244	Abkhaz	Northwest Caucasian	(Q) Hewitt 1989: 157, 159
basq1248	Basque	Basque	(Q) Saltarelli 1988: 210; Trask 2003: 122; de Rijk 2008: 482, 501f.; Areta 2009: 67; Artiagoitia 2012
clas1250	Classical Nahuatl	Aztecan	Andrews 1975: 192–194; Andrews 2003: ch. 17.3
even1259	Evenki	Tungusic	(Q) Nedjalkov 1997: 197, 199
hixk1239	Hixkaryana	Cariban	(Q) Derbyshire 1979: 131
nucl1643	Japanese	Japanese	(Q) Hinds 1988: 254, 261; Noguchi 1997: 780; Furuya 2008: sec. 3.2; Inokuma 2009
kore1280	Korean	Korean	(Q) Sohn 1994: 284; 292; Choi 2014: 151–154
kala1399	Kalaallisut (W. Greenlandic)	Eskimo	(Q) Fortescue 1984: 110, 253, 256f.
lakk1238	Lakkia	Kadai	Fan 2019: 137
nucl1301	Turkish	Turkic	(Q) Kornfilt 1997: 288, 297f.

5 Annotation scheme of csv

language language name
glottolog glottocode following glottolog (Hammarström et al. 2021)
sortclass manual coding of phylogenetic information purely for more accessible sorting of data presentation
family language family based on glottolog (Hammarström et al. 2021)
genus.print genus information for presentation purposes
genus.WALS genus following WALS (Dryer &Haspelmath 2013) where available, otherwise manually supplied

printname language name used on world map
Oc.printname language name used on maps for Oceania
print set to 1 if language printed on map
lat geographical coordinates based on WALS and glottolog, latitude
lat geographical coordinates based on WALS and glottolog, longitude
hjust horizontal adjustment for printing language name on world map
vjust vertical adjustment for printing language name on world map
Oc.hjust horizontal adjustment for printing language name on map for Oceania
Oc.vjust vertical adjustment for printing language name on map for Oceania
area.glottolog area information based on glottolog (Hammarström et al. 2021)
area.WALS area information based on WALS (Dryer &Haspelmath 2013)
ref.NomPers bibliographic information on PERS$_N$; (Q) indicates questionnaire-based Routledge grammars
NomPers value **y** iff information on PERS$_N$ available; **n** if not
APC value **y** iff APCs attested; **n** if not
WO.WALS verb-object order based on Dryer (2013d)
WO data from WO.WALS and manually supplied values for gaps
adpos.manual manually collected adposition-noun order data
adpos.WALS adposition-noun order based on Dryer (2013a)
adpos data from adpos.WALS and manually supplied values for gaps
genitive.WALS genitive-noun order based on Dryer (2013c)
genitive data from genitive.WALS and manually supplied values for gaps
DemDir.WALS demonstrative-noun order based on Dryer (2013b)
DemDir.manual manually collected demonstrative-noun order data
DemDir data from DemDir.WALS and manually supplied values for gaps
genitive.WALS genitive-noun order based on Dryer (2013c)
genitive data from genitive.WALS and manually supplied values for gaps
ArtOrder order of article and noun if available, **NA** if no articles
Art.with.Nompers value **y** iff articles attested in APCs, **n** if not possible, **unclear** if unknown, **NA** if language without articles
APC.dir value **pre** iff prenominal APCs, **post** for postnominal APCs, **both** for ambidirectional APCs, **apc** if no APCs attested
boundPers value **pre** iff prenominal BPCs, **post** for postnominal BPCs, **n** if no bound PERS$_N$-marking
boundPers.simultAPC value **pre** if prenominal APC-like marking attested simultaneously with bound PERS$_N$-marking, **post** if postnominal APC-like marking attested simultaneously with bound PERS$_N$-marking, **NA** otherwise
PPDC value **y** iff PPDCs attested; **y-nn** if attested PPDCs include only demonstrative and PERS$_N$-marking, but no lexical material; **NA** if not attested
PPDC.ref bibliographic references for PPDCs

6 List of examples

6.1 Afroasiatic languages

6.1.1 Hausa (haus1257), West Chadic

(14) a. *mū Háusàwā*
we Hausa
'we Hausa'
(Newman 2000: 371)

b. *shī wannàn mālàmī*
he DEM.1 teacher
'he (this) teacher'
(after Newman 2000: 371)

c. *mū màlàman-nàn*
we teacher-DEM.PROX
'we these

See Newman (2000: 63, 155, 370f.) and also Jaggar (2001: 330f.) for further examples.

6.1.2 Mupun (mwag1236), West Chadic

(15) *war manaja nə*
3F DEF
'she, the manager'
(Frajzyngier 1993: 172, (154))

6.1.3 Gorwaa (goro1270), Southern Cushitic

(16) *atén oo hhawató*
Pro1PL ANAPH.M men.LNK.M
'we men'
(after Harvey 2018: 163, (2.205))

6.1.4 Kambaata (kamb1316), East Cushitic

(17) *na'óot* *Kambáat-u*
1PL.NOM Kambaata-M.NOM
'we Kambaata (people)'
(Treis 2008: 335, (1097))

6.1.5 Cairene Egyptian Colloquial Arabic (egyp1253), Semitic

(18) *ʔintu* *ʔit[-]talamza* *tiħibbu* *ʔilliʕb*
you.PL [DEF-]students 2PL.like playing
'You students like playing.'
(Gary &Gamal-Eldin 1982: 80, (533))

See Gary &Gamal-Eldin 1982: 78; 80.

6.1.6 Gulf Arabic (gulf1241), Semitic

(19) *iHna* *T-Tullaab* *ma* *nigdar* *nigbal* *haadha* *l-qaraar*
we DEF-students not 1PL-be-able 1PL-accept this DEF-decision
'We students cannot accept this decision'
(Holes 1990: 165, (845))

See Holes 1990: 162, 165.

6.1.7 Maltese (malt1254), Semitic

(20) *[Intom* *il-ħaddiema]* *għandkom* *tingħaqdu* *[Maltese]*
you DEF-workers have.2PL unite.2PL
'You workmen should unite together.'
(Borg &Azzopardi-Alexander 1997: 202, (915))

6.2 Australian languages

6.2.1 Mangarrayi (mang1381), Mangarrayi

(21) *ŋjḷa* *malam-gaḷa* *ga-ŋiḷa-ṇi* *ṇa-waḷayjñin-gan*
1pl.excl.nom man-pl.nom 3-1pl.excl-sit NLoc-shade
'We men are sitting in the shade.'
(Merlan 1989: 103)

See Merlan 1989: 103; 203 for discussion.

6.2.2 Diyari (pirl1241), Central Pama-Nyungan

(22) a. *ngayani* *waka-li* *thananha* *nhayi-yi*
1pl.excl.nom small-erg 3pl.accsee-prs
'We small (ones) watch them'
(Austin 2013: 102, (106))

b. *ngarda-nhi* *thana-li* *nhinha* *yakalka-yi* *yaru-ya*
then-loc 3pl.erg 3sg.nf.accask-prs like that-prox
wardayari-lha *yini* *pinarru*
where-char sg.nom old
'Then they asked him the following: "Where are you from, old man?" '
(Austin 2013: 119, (175))

c. *nhani* *mankarra* *thurara-yi* *thana-ngu-ya* *kinthala-nhi*
3sg.f.nom girl.nom lie-prs 3pl-loc-prox dog-loc
'The girl is lying with those dogs'
(Austin 2013: 150, (311))

For discussion and more examples see Austin (1981: 97f.) and (Austin 2013: 100–103, 119, 150).

6.2.3 Warlpiri (warl1254), Western Pama-Nyungan

(23) a. *ŋarka* *njanuŋu* *ka* *puḷa-mi*
man 3 prs shout-npst
'The aforementioned man is shouting'
(Hale 1973: 316, (22))

b. *yapa ŋatju*
person 1SG
'I person'
(Hale 1973: 317)

See also Reece 1970: 70 and especially Hale 1973: 316f. for further discussion.

6.2.4 Pitjantjatjara (pitj1243), Western Pama-Nyungan

(24) a. *Minyma palur̠u ngayu-nya nya-ngu*
woman 3SG.NOM 1SG.ACC see-PST
'The woman saw me.'
(Bowe 1990: 31, (110))

b. *Palur̠u wati nyara war̠a-ngku mutaka palya-nu*
3SG.NOM man distant tall-ERG car fix-PST
'The tall man over there (in contrast to the other one) fixed the car.'
(Bowe 1990: 34, (114))

See Bowe (1990: 49–51) for discussion.

6.2.5 Guugu Yimidhirr (gugu1255), Northern Pama-Nyungan

(25) a. *Nyulu nhayun waarigan gada-y waanggu=wunaarna-y.*
3SG.NOM that.ABS moon.ABS come-PST sleep=lie+REDUP-PST
'[Then] the Moon came and lay down to sleep.'
(Haviland 1979: 157, (423))

b. *Bidha nyulu biini.*
child.ABS 3SG.NOM die.PST
'The child died.'
(Haviland 1979: 157, (424a))

For general description suggesting that "the norm arrangement for an NP that refers to a human" involves a prenominal APC see Haviland (1979: 104).

6.2.6 Kuku Yalanji (kuku1273), Northern Pama-Nyungan

(26) a.

nyulu	*jalbu*
3SG	woman

‘**the** woman’
(after Patz 2002: 202; gloss extrapolated)

b.

Yurra	*karrkay*	*dunga-y*	*bana*	*mana!*
2PL.NOM(S)	child.ABS(S)	go-IMP	water.ABS(OBJ)	get.IMP

‘You children go and get water!’
(after Patz 2002: 203, (620))

(27) a.

Pastor	***nyulu****…*
pastor	3SG

‘Pastor, he…’
(after Patz 2002: 202, (611); gloss extrapolated)

b.

Ngayu	*babi*	*wilbuman*	*yindu*
1SG.NOM(S)	father’s.mother.ABS(S)	old.woman.ABS(S)	other.ABS(S)

ngamu	***nganjin***	*dunga-ri-ny*	*mayi*	*baka-nka.*
mother.ABS(S)	1PL.EXCL.NOM(S)	go-PL-PST	food.ABS(OBJ)	dig-PURP

‘I, grandmother, another old woman and mother, we went out to dig for food (yams).’
(after Patz 2002: 203, (618))

See Patz (2002: 120f., 202f.).

6.2.7 Kayardild (kaya1319), Tangkic

(28)

niya	*jungarra*	*dangkaa*
he	big	man

‘the big man’
(Evans 1995: 239)

See also Evans (1995: 239; 251) and Round (2013: 141).

6.3 Austronesian languages

6.3.1 Malagasy (mala1537), Barito

(29) *izaho* *vehivavy*
1SG.DEF woman
'I woman'
(Paul &Travis 2019: 411, (6a))

For further discussion of some complex variation patterns see Paul &Travis (2019).

6.3.2 Indonesian (stin1234), Malayo-Sumbawan

(30) *kami,* *bangsa* *Indonesia*
we people Indonesia
'we, the people of Indonesia'
(Sneddon 1996: 170)

6.3.3 Papuan Malay (papu1250), Malayo-Sumbawan

(31) *de* *blang,* *a,* ***om*** ***ko*** ***ini*** *tra* *liat…*
3SG say ah! uncle 2SG DEM.PROX NEG see
'he said, "ah, **you uncle there** didn't see…" '
(Kluge 2017: 353, (66))

See Kluge (2017: ch. 6.2) for detailed discussion and more examples.

6.3.4 Loniu (loni1238), Oceanic

(32) a. *sɛh* *pihin* *sɛh* *čani* *uwɛh* *kaman* *uwɛh* *wɛčɛ* *ake*
3PL woman 3PL clear 1PL.EXCL man 1PL.EXCL cut.down tree
'The women clear, we men cut down the trees.'
(Hamel 1994: 90, (6))

b. *iy* *amat* *iyɔ*
3SG man DEM
'this man'
(Hamel 1994: 100, (89))

See Hamel (1994: sec. 4.2.1) for discussion.

6.3.5 Windesi Wamesa (wame1241), Oceanic

(33) *sinitu=pa-**tata*** [Windesi Wamesa]
person=DET-1PL.INCL
'we people'
(Gasser 2014: 144, (3.46))

6.3.6 Maori (maor1246), Oceanic

(34) *E kaha rawa atu [maatou ngaa kaiako naa] ki te pata·patai*
TAM strong very away 1PL.EXCL the.PL teacher DEM.2 to the REDUP·ask
'We teachers ask a lot of questions.'
(Bauer 1993: 373, (1673))

For discussion and more examples see Bauer 1993: 368, 373 and Bauer 1997: 262f..

6.3.7 Tuvaluan (tuva1244), Oceanic

(35) a. *Au ttino poto koo leva ne iloa nee au mea kolaa*
I the+person intelligent PFV know ERG I thing those
faatoaa iloa nee koe ttagata valea.
just know ERG you the+man stupid
'I, an intelligent person, have long known what you, stupid man, are just discovering.'

b. *Taatou tino Tuuvalu e see tau ki meakkai kolaa.*
we.INCL person Tuvalu NPST NEG befit to food those
'We Tuvaluans are not accustomed to that [type of] food.'
(Besnier 2000: 393, (2018/2019))

See Besnier 2000: 392f..

6.3.8 Kwaio (kwai1243), Oceanic

(36) a. *'a-gauru-a ta'a i 'Ai'eda*
FPRON.3TRI-? people LOC 'Ai'eda
'those 'Ai'eda people'

b. *fa-meru-a ta'a geni*
for-SPRON.1TRI.EXCL-? people female
'for us women'
(after Keesing 1985: 104)

The status of the *-a* intervening between pronoun and noun is not clear, see Höhn (2020: 25) for speculation that this might be a reduced article.

6.3.9 Arop-Lokep (arop1243), Oceanic

(37) *am garup ke Bok*
1EXCL.PL female.one of Bok
'we women of Bok village'
(D'Jernes 2002: 255)

6.3.10 Cheke Holo (chek1238), Oceanic

(38) a. *Tahati naikno ḡre e kmana pui puhi=da*
1PL.INCL people DEM.PROX.PL EMPH lot.of DUR way=1PL.POSS
'We people have had many problems.'
(Boswell 2018: 165, (591))

b. *Gotilo Honiara fa-le lehe egu*
2PL Honiara CAUS-DUR die like.that
'You people of Honiara kill [people], like that.'
(Boswell 2018: 99, (301))

6.3.11 Hoava (hoav1238), Oceanic

(39) *Maki lavati sa pa Solomone, gi ta-n̠ani gita nikana hupa.*
NEG.IMP big 3SG LOC Solomons and PASS-eat 1PL.INCL man black
'Let it [a monkey] not grow big in the Solomons, and we black men be bitten.'
(after Davis 2003: 48, (72a))

See Palmer 2017 for detailed discussion.

6.3.12 Kokota (koko1269), Oceanic

(40) a. *gai nakoni zuzufra*
we.EXCL person black
'we black people'
(Palmer 2008: 95, (3.70a))

b. *ka gai ira nakoni zuzufra tana nogoi naito tahi ke*
LOC we.INCL the.PL person black then VOC devil sea PFV
age̅=u=ni=u
go=be.thus=3SG.OBJ=CNT
'With us black people, then, man!, 'sea devil' is what it's called.'
(Palmer 2008: 123, (4.1b))

See Palmer (2008: 68, 95, 116, 119, 123, 131, 137, 163, 242, 300, 305, 327, 399, 414) for various examples.

6.4 Dravidian languages

6.4.1 Kannada (nucl1305), Dravidian

(41) *na:vu bra:hmaNaru vidyeyannu mareyaba:radu.*
we Brahmin-PL education-ACC forget-INF-PROH
'We Brahmins shouldn't forget education.'
(Sridhar 1990: 209, (755))

See Sridhar (1990: 205, 208f.).

6.4.2 Malayalam (mala1464), Dravidian

(42) *ɲaŋŋaḷ intyakkaar adhikavum adhikavum sasyabhuukkukaḷ aaṇə*
we.EXCL Indians mostly vegetarian-PL be-PRS
'We Indians are mostly vegetarians.'
(Asher &Kumari 1997: 262, (1340))

See Asher &Kumari 1997: 262f. for more examples.

6.4.3 Tamil (tami1289), Dravidian

(43)

naaŋka	*iŋliişkaaraŋkellaam*	*kaaramaana*	*vastu*	*caappiʈaratille*
we	English-person-PL-all	spicy	thing	eat-PRS-NOM-NEG

‘We English do not eat hot things.’
(Asher 1985: 146)

See Asher (1985: 142, 146).

6.5 Indo-European languages

6.5.1 Welsh (wels1247), Celtic

(44)

ni	*fyfyrwyr*
we	SOFTMUT.student.PL

’we students’
(David Willis, personal communication)

6.5.2 Danish (dani1285), Germanic

(45)

lad	*os*	*voksne*	*snakke*	*i*	*fred*
let.IMP	1.PL.ACC	adults	talk.INF	in	peace

‘Let us adults talk in peace.’
(Schröter 2021: 29, (32b))

See Schröter (2021) for detailed discussion and Johannessen (2008) for psychologically distant demonstratives, corresponding to adnominal third person singular pronouns.

6.5.3 Icelandic (icel1247), Germanic

(46)

við/	*þið*	*Íslendigar*
we	you.PL	Icelanders

‘we/ you Icelanders’
(Sigurðsson &Wood 2020: 11, (16a))

See Sigurðsson &Wood (2020: 11f.) for further examples and Johannessen (2008) for psychologically distant demonstratives, corresponding to adnominal third person singular pronouns.

6.5.4 Norwegian (norw1258), Germanic

(47) *til oss (to) (gaml-e) professor-a-ne*
to us two old-DEF professor-PL-DEF
'to us (two) (old) professors'
(Julien 2005: 129, (4.36))

See also Johannessen (2008) for psychologically distant demonstratives, corresponding to adnominal third person singular pronouns.

6.5.5 Swedish (swed1254), Germanic

(48) *vi student-er*
we student-PL
'we students'
(Julien 2005: 128, fn. 18, (ia))

See also Johannessen (2008) for psychologically distant demonstratives, corresponding to adnominal third person singular pronouns.

6.5.6 Dutch (dutc1256), Germanic

(49) *Wij/ jullie taalkundigen denken te veel na.*
we you.PL linguists think too much PRTCL
'We/you linguists think too much.'
(Corver 2008: 52, (23a))

6.5.7 English (stan1293), Germanic

(50) you linguists

See among others: Delorme &Dougherty (1972); Keizer (2016); Pesetsky (1978); Postal (1969); Sommerstein (1972).

6.5.8 German (stan1295), Germanic

(51)

Wenn	*noch*	*nicht*	*einmal*	*[du*	*Linguist]*	*die*
if	still	NEG	PRTCL	you.SG	linguist	DET.ACC.SG

neue	*Rechtschreibung*	*beherrschst…*
new	orthography	command.2SG

'If not even you linguist have a command of the new orthography rules…'
(after Rauh 2004: 100, (36))

See among others: Lawrenz (1993: ch. 6), Rauh (2003, 2004) and Roehrs (2005).

6.5.9 Standard Modern Greek (mode1248), Greek

(52)

Emis	*i*	*glosoloji*	*piname.*
we	DET.PL	linguists	are.hungry.1PL

'We linguists are starving/hungry.'
(after Lekakou &Szendrői 2012: 114, (12c))

See Stavrou (1995) as well as Choi (2014: chs. 1/2) and Höhn (2016: sec. 5).

6.5.10 Calabrian Greek/Greko (apul1236), Greek

(53)

Emì	*ta*	*pedìa*	*den*	*pìnnome*	*kafè.*
we	DET.N.PL	children.N	NEG	drink.1PL	coffee

'We children don't drink coffee.'
(after Höhn et al. 2017: 274, (17))

See Höhn et al. (2017) for further discussion.

6.5.11 Kashmiri (kash1277), Indic

(54) *əs' kə:shir' chi kə:r'gar.*
we Kashmiris are artisans
'We Kashmiris are artisans.'
(Wali &Koul 1997: 200, (8))

See Wali &Koul (1997: 200).

6.5.12 Marathi (mara1378), Indic

(55) *āmhī bāyakā nehmīts āplyā kutumbā̃n sāṭhī khapto*
we women always-emph refl families for work.hard-1pl
'We women always work hard for the sake of our families.'
(Pandharipande 1997: 386, (1141))

See Pandharipande (1997: 381, 386) for more examples.

6.5.13 Punjabi (panj1256), Indic

(56) *asī̃ pañjaabii garam mijaaj de ãã.*
we Punjabi hot nature gen.m.pl are
'We Punjabis are hot blooded/tempered.'
(Bhatia 1993: 228, (703))

See Bhatia (1993: 228).

6.5.14 Persian (west2369), Iranian

(57) *ma irani-a*
we Iranian-pl
'we Iranians'
(Mahootian 1997: 212, (356))

See Mahootian (1997: 209, 212).

6.5.15 Aromanian (arom1237), Romance

(58) *noi pikurar-li adrem pini.*
we shepherd-DEF.PL baled bread
'We shepherds baked bread.'
(elicited)

See also Höhn (2016: 560).

6.5.16 Romanian (roma1327), Romance

(59) *Voi avocații vă apărați clienții.*
you.PL lawyers-DEF CL.2PL defend cuzstomers-DEF
'You lawyers defend your clients.'
(Cornilescu &Nicolae 2014: 10, (20a))

For further examples see Mallinson (1986: 255, 258) and Cornilescu &Nicolae (2014: 6, 10, 20f.).

6.5.17 Catalan (stan1289), Romance

(60) *nosaltres els bombers*
we DET.PL firemen
'we firemen'
(Hualde 1992: 290)

See also Hualde (1992: 287, 290) and Höhn (2016: 560).

6.5.18 Galician (gali1258), Romance

(61) *nos os estudantes*
we DET.PL students
'we students'
(Höhn 2016: 560, (36a))

See Höhn 2016: 560, also Álvarez et al. (1986: 152, 301).

6.5.19 European Portuguese (port1283), Romance

(62)

Nós	*portugueses*	*bebemos*	*bom*	*café.*
we	Portuguese	drink.1PL	good	coffee

‘We Portuguese drink good coffee.’
(Höhn 2016: 555)

6.5.20 Spanish (stan1288), Romance

(63)

Nosotros	*los*	*lingüistas*	*somos*	*listos.*
we	DET.PL	linguists	be.1PL	smart

‘We linguists are smart.’
(Choi 2014: 210, (38a))

See Choi (2014: 210f.) and Höhn (2016: 560) as well as de Bruyne (1995: 145).

6.5.21 Italian (ital1282), Romance

(64)

noi/	*voi*	*linguisti*
we	you.PL	linguists

‘we/you linguists’
(Cardinaletti 1994: 202, (21a))

See Cardinaletti (1994: 202f.), Höhn (2016: 559) and also Höhn et al. (2016, 2017).

6.5.22 Northern Calabrese (nort2612), Romance

(65)

Nua	*i*	*quatrarə*	*iucamə*	*i*	*cartə.*
we	DET.PL	children	play.1PL	DET.PL	cards

‘We children play cards.’
(Höhn et al. 2017: 276, (23b))

See similarly Höhn et al. (2016: 142).

6.5.23 Southern Calabrese (sout2616), Romance

(66)

Nui	*i*	*figghioli*	*iocamu*	*e*	*carti.*
we	DET.PL	children	play.1PL	DET.PL	cards

‘We children play cards.’
(Höhn et al. 2017: 276, (23c))

See similarly Höhn et al. (2016: 142)

6.5.24 Russian (russ1263), Slavic

(67)

my	*leningradcy*
we	Leningrader.PL

‘we Leningraders’
(Pesetsky 1978: 352, (4c))

6.5.25 Bulgarian (bulg1262), Slavic

(68)

nie	*student-i-te*
we	students-PL-DEF

‘we students’
(Höhn 2016: 560)

See also Osenova (2003) and Norman (2001) for discussion on unagreement phenomenon in Bulgarian.

6.5.26 Pomak (poma1238), Slavic

(69)

’nami	*Po’matsem-se*	*no*	*na*	*pʌ’maga*	*’nikutri*
we.DAT	Pomak.DAT.PL-DET.PROX	1PL.ACC	NEG	help.3SG	nobody

‘Nobody helps us Pomaks.’
(Papadimitriou 2008: 582)

See also Höhn (2017: 269–272) for some related discussion.

6.5.27 Polish (poli1260), Slavic

(70) *My lingwiści lubimy formalizację.*
we linguists like.1PL formalisation
‘We linguists like formalisation.’
(Rutkowski 2002: 161f., (3b))

6.6 Khoe-Kwadi languages

6.6.1 Khoekhoe/Nama (nama1264), Khoe-Kwadi

(71) *sa k͜hoe-ta ké nĩ ra ||’o.*
ART.ADDR person-1PL.INCL.CG TOP? COMPEL PROG die
‘We humans have to die.’
(after Böhm 1985: 133, (27b))

See Böhm (1985: 133–145) and Maho (1998: 140) and particularly Haacke (2013, 1976, 1977) for discussion.

6.6.2 Khwe/Kxoe (kxoe1243), Khoe-Kwadi

(72) *Hè é tó Khwé-tò dì góɛ́ à tó ò*
DEM 2PL.CG Khwe-2PL.CG POSS cattle OBJ 2PL.CG POSS
||é Qúva-||è ǂxà-á-tè à.
1PL.M White-1PL.M give-I-PRS OBJ
‘Here are yours, the Khwe’s cows that we, the Whites, give you.’
(Kilian-Hatz 2008: 41, (1) quoting Köhler 1989: 514f.)

See Kilian-Hatz (2008: ch. 3.1.2) for discussion and Kilian-Hatz (2008: 79) for a further example.

6.7 Niger-Congo languages

6.7.1 Kinyarwanda (kiny1244), Bantu

(73) *N-ubah-a wow mu-kire.*
1SG.SBJV-respect-FV 2SG NCL1-rich.man
‘I respect you rich man.’
(van der Wal 2022: 69, (73a))

See van der Wal (2022: 67–69) for discussion.

6.7.2 Luganda (gand1255), Bantu

(74)

Ffe	*abantu*	*abaavu*	*ffe tubonaabona.*
we	people	poor	1PL.suffer

‘We poor people suffer.’
(elicited)

See also Ashton et al. (1954: 102) for further (unglossed) examples.

6.7.3 Nkore-Kiga (nkor1241), Bantu

(75)

itwe	*abanyankore*	*ni-tu-hinga*	*ebinyoobwa*
we	Banyankole	PRS.IPFV-1PL-cultivate	groundnuts

‘We Banyankole grow groundnuts.’
(after Taylor 1985: 131, (368))

6.7.4 Nzadi (nzad1234), Bantu

(76)

ntə̂m.	*yɛ*	*kó*	*luzíŋ*	*↓é*	*bï*	*andzéé*	*bǐ*	*k*	*yéè*	*ntswé*	*ninyá*
taste	and	LOC	life	of	us	Nzadi.people	we	NEG.PRS	sell	fish	that

bɔ.	*atá[…]*
NEG	even

‘…tasty. In the tradition of us Nzadi, we don’t sell that fish. Even…’
(Crane et al. 2011: 279, (10))

See Crane et al. (2011: 210, 279).

6.7.5 Swahili (swah1253), Bantu

(77)

Nyinyi	*wa-nafunzi*	*m-me-cheka.*
you.PL	NCL2-student	2PL-PST-laugh

‘You students laughed.’
(elicited)

Concerning unagreement (Ackema &Neeleman 2013; Hurtado 1985) in Swahili see Höhn (2016: 546) for a short note.

6.7.6 Babungo (veng1238), Wide Grassfields

(78) *yìa* *v*ɨ́ɨ *ndâa* *gə́* *ntó'*
we.EXCL people smithy go-PRS palace
'We, the blacksmiths, go to the palace.'
(Schaub 1985: 197, (134a))

See Schaub (1985: 197f.) for discussion.

6.7.7 Koromfe (koro1298), Koromfe

(79) *ʋkɔ* *(a)* *korombʌ*
we ART Koromba
'we Koromba'
(Rennison 1997: 251, (585)))

The article may be dropped in fast speech, for more discussion see Rennison (1997: 242, 250f.).

6.7.8 Supyire (supy1237), Senufo

(80) *wùu* *shìin* *taanré*
we person.NCL1.PL three
'we three'
(after Carlson 1994: 208, (45a))

See Carlson 1994: 207f..

6.8 "Papuan" languages (non-Austronesian languages of Oceania)

6.8.1 Menya (meny1245), Nuclear Angan

(81) a. *Nyi tä=ŋga=ŋi Matiu i=qu=k=i kukŋuä* *[Menya]*
1SG this=TIME=GVN Matthew DEM=M=**2SG**=OBJ talk

hn=i yatŋqä k-i-m=ŋqä=i.
INDF=F ask 2SG-do-1SG/IRR=GOAL=IND

'I'm now going to ask you Matthew something.'

b. ***Ne*** *ämaqä qokä i=qu=ne yiämisaŋä huiyi=nä qw*
1PL person man DEM=M=**1PL** food other=FOC CERT

ä-n-k-qäqu=i.
ASS-eat-PST/PFV-1PL/DSO=IND

'Then we men ate some other food.'
(Whitehead 2006: 30, (58/59))

Possible person marking on an indefinite expression:

(82) *Hn=qu=ki quwä ä-ma-t-qä=i*
INDF=M=2SG steal ASS-get-2SG/IRR-GENER=DEF

'Should one of you steal something…'
(Whitehead 2013: 9, (18))

See Whitehead (2006: 40, 46, 56f.) and Whitehead (2013: 9f., 18f.) for further examples and discussion.

6.8.2 Momu (fass1245), Baibai-Fas

(83) *Yery mu, baso nenwu wu-ta-r-u.*
1PL women child belly INAN:be.at-STVZR-1PLSG-NMLZ

'For we women, we get pregnant (lit. children are in our bellies).'
(Honeyman 2016: 568, (4))

See Honeyman (2016: 169f., 242, 568).

6.8.3 Imonda (imon1245), Border

(84) *ka sebuhe tõgõ fi-li-t*
1 devil thus do-EMPH-CF

'We devils should have done it like that.'
(Seiler 1985: 61, (8))

See Seiler (1985: 44, 61f.). The possibility for postnominal APCs is mentioned, but unfortunately no example is provided.

6.8.4 Bilua (bilu1245), Bilua

(85) a. ***enge****=a* *Solomoni=a=ma* *maba* *poso=****ngela***
1PL.EXCL=LIG Solomon=LIG=3SG.F person PL.M=1PL.EXCL
'we, Solomon people'
(Obata 2003: 85, (7.35))

b. … *lai=za=****mu****=****mela*** *inio* *me.*
where=LIG=3PL=2PL FOC.NF 2PL
'…you are people from where?'
(Obata 2003: 88, (7.49))

See Obata (2003: 47–49, 76, 79, 84f., 87–89, 92f.) for a range of further examples.

6.8.5 Lavukaleve (lavu1241), Lavukaleve

(86) *aka* ***malav*** ***e*** *roa-ru* *kiu-la-m.*
then people 1PL.EXCL one.SGM-none die-NEG-SGM
'And we, the people [lit: the people we] didn't die. [i.e. None of us people died.]'
(after Terrill 2003: 171, (197))

See Terrill (2003: 171–173).

6.8.6 Moskona (mosk1236), East Bird's Head

(87) a. ***mi****-osnok* *mi-en-ah-miy,* *mi-en-ot* *jig* *miyes*
1PL-person 1PL-DUR-bathe 1PL-DUR-stand LOC clothes
mi-er *tofi.*
1PL-wear hat
'we people bathe, wear clothes, wear hats…' (stand in clothes = wear clothes)
(Gravelle 2010: 344, (2))

b.

Eri	***i**-ejen(a)*	*i-odog*	*jig…*
they.PL	3PL-woman	3PL-pregnant	LOC

'(If/when) they the women are pregnant…'
(Gravelle 2010: 91, (38))

c.

eri	*Mosmir*
they	Maybrat

'Maybrat people/tribe'
(Gravelle 2010: 224, (43b))

See Gravelle (2010: 91, 222–224, 344).

6.8.7 Sougb (mani1235), East Bird's Head

(88)

Emen	*Sougb/*	*emen*	*meijouhw*	*dangga.*
we.EXCL	Sougb	we.EXCL	custom	like.that

'We Sougb, that's our custom.'
(Reesink 2002: 274, (40))

See Reesink (2002: 200, 269f., 274).

6.8.8 Hatam (hata1243), Hatim-Mansim

(89)

Nye-ni	*sop*	*Tinam*	*hwop*	*yok*	*nya*	*ci*	*gi-mbres#*
we-this	woman	Tinam	girl	put	PL	chase-away	NMLZ-wide

'We Tinam women are not easy to take.'
(Reesink 1987: 195, (72))

6.8.9 Maybrat (maib1239), Maybrat

(90)

fnia	*anu*	*p-no*	*po*	*re-t-o*	*fawen*	*fe*
woman	2PL/1PL.INCL	1PL-do	thing	location.SPEC-PROX-U	long.time	NEG

'We women (inc), we haven't done this thing for a long time.'
(Dol 2007: 158, (99))

See Dol (2007: 64, fn. 5) concerning the 1PL.INCL use of the pronoun *anu.* See Dol (2007: 141, 158, 172, (281)) for examples.

6.8.10 Urim (urim1252), Urim

(91) *men* *melnum* *watipmen*
1PL.EXCL person many
'we(excl.) many people'
(Hemmilä &Luoma 1987: 123)

(92) a. *tu* *melnum*
3PL person
'the people'

b. *kupm* *Melming* *la* *nak-etn* *por* *ti*
1SG Melming say tell.REAL-2SG.OBJ story this
'I Melming told you this story.'
(Hemmilä &Luoma 1987: 125)

See Hemmilä &Luoma (1987: 123, 125).

6.8.11 Savosavo (savo1255), Savosavo

(93) *[[]No* ***mapa=gha]**NP* *[**ave**]NP* *]NP=na* *kula* *ata*
2SG.[GEN] person=PL 1PL.EXCL=NOM seawards here 2SG-GEN.M
no-va *nito=la.*
eye=LOC.M
'[Addressing the volcano:] We, your people, (are) here seawards at your eye.'
(Wegener 2012: 155, (285))

See Wegener (2012: 147,155–159).

6.8.12 Manambu (mana1298), Sepik, Ndu

(94) *wun* *ñən-a* *ñaməs*
I you.F-LNK+F.SG younger.sibling
'me, your younger sister'
(Aikhenvald 2008: 197)

See Aikhenvald (2008: 197f., 508–513) for further examples and discussion.

6.8.13 Awtuw (awtu1239), Sepik, Ram

(95) a. *rom* *Meley-yænim*
3PL Meley-GENER
'the people from Meley'
(Feldman 1986: 122, (15a))

b. *rey/tey* *tale*
3M.SG/3F.SG woman
'the woman'
(Feldman 1986: 123, (21a))

See Feldman (1986: 120–124) for discussion.

6.8.14 Alamblak (alam1246), Sepik, Sepik Hill

(96) a. *yima-**m***
person-3PL
'people'
(Bruce 1984: 96)

b. *yima-**kë***
person-2PL
'you people'

c. *yima-**nëm***
person-1PL
'we people'

See Bruce (1984: 90–92, 96f.).

6.8.15 Fore (fore1270), TNG, Fore-Gimi

(97) *aogi* *yagara:'-na:* *kana-u-e*
good man-1SG come-1SG-IND
'I, the good man, come.'
(after Scott 1978: 80)

See Scott (1978: 79f., 100f.).

6.8.16 Hua (huaa1250), TNG, Siane-Yagaria

(98) a. *Forapi' + da → /forapi da/* 'I, Forapi'

b. *Forapi' + Ka → /forapiga/* 'You, Forapi'

c. *nono' + 'Kama' + da → /nonokama da/* 'I your maternal uncle'
(Haiman 1980: 226)

(99) Person marked genitive forms(after Haiman 1980: 240)

a. vimata
'of us men'

b. ademata
'of us women'

c. vi'ita
'of you men'

d. adita
'of you women'

See Haiman (1980: 226–232, 239f.).

6.8.17 Yagaria (yaga1260), TNG, Siane-Yagaria

(100) *dagaea ve* ***agaea*** *∅-begi-d-u-e*
I man he him-hit-PST-1SG-IND
'I hit the man.'
(Renck 1975: 18f.)

(101) a. *Ovu-**da** ma-lo' bei-d-u-e*
Ovi-I this-LOC live-PST-1.SG-IND
'I, Ovu, am here.'

b. *a-**tata** e-d-a'-e*
woman-they.DU come-PST-3.DU-IND
'The two women came.'
(Renck 1975: 19)

See Renck (1975: 17f., 166, 181).

6.8.18 Amele (amel1241), TNG, Mabuso

(102) *[Dana ben eu age] ho-ig-a.*
man big that 3PL come-3PL-TODPST
'Those leaders (big men) came.'
(after Roberts 1987: 210, (283)-(284))

See Roberts (1987: 162, 201, 209f.).

6.8.19 Kobon (kobo1249), TNG, Kalam-Kobon

(103) a. *Juab Minöp kalɨp ñ-öb.*
Juab Minöp OBJ.3DU give-PRF.3SG
'He gave it to Juap and Minöp.'
(after Davies 1989: 108, (264))

b. *Yad Kaunsol nibi bɨ abad aij gɨ-mɨd-pin.*
1SG councillor woman man look after good do-HABIT-PFV.1SG
'As councillor I look after the people well.'
(Davies 1989: 157, (408ad))

See Davies (1989: 107f., 157) for instances of prenominal pronouns as well as postnominal "pronominal copies".

6.8.20 Usan (usan1239), TNG, North Adelbert

(104) a. *eâb igim-ine ne tain [**wo**] yâ-nâmb wogub…* *[Usan]*
cry.SS be-1SG.DS and father he me-hit.SS cease.SS
'I was crying and my father he hit me and then…'
(Reesink 1987: 167, (99))

b. … *in bo **an** wau moi qomon gâs ende ig-oun*
we again you child unmarried custom like thus be-1PL.PRS
'…we in turn live like the customs of you young men.'
(part of Reesink 1987: 190f., (41))

See Reesink (1987: 53f., 167, 190f., 353).

6.8.21 Adang (adan1251), TNG, Alor-Pantar

(105) *Sa* *[Bain ʔari]*$_{NP}$ *bɛh.*
3SG.SBJ Bain 3.OBJ hit
'S/he hit Bain.'
(Robinson &Haan 2014: 261, (165))

6.8.22 Kaera (kaer1234), TNG, Alor-Pantar

(106) *Ilwang gang user~user bir bleling g-om mi eser-o.*
Ilwang 3SG REDUP~quickly run open 3SG-inside LOC exit-FIN
'Ilwang quickly ran outside.' (lit. '…ran out to (the) open's inside')
(Klamer 2014: 129, (98))

6.8.23 Kamang (kama1365), TNG, Alor-Pantar

(107) *almakang=ak gera*
people=DEF 3.CONTR
'the {specific group of} people'
(Schapper 2014: 313f., (58a))

See Schapper (2014: 313f.).

6.8.24 Sawila (sawi1256), TNG, Alor-Pantar

(108) *[aning du girra]*$_{A}$ *[parra]*$_{P}$ *laata*
NFIN.person PL 3.A field burn
'People are burning fields.'
(Kratochvíl 2014: 392, (102f))

See Kratochvíl 2014: 391f..

6.8.25 Wersing (wers1238), TNG, Alor-Pantar

(109) *aning* ***gnuk*** *unan* *le-wena*
person 3.du louse appl-search
'Those two people searched for lice.'
(Schapper &Hendery 2014: 472, (75))

See Schapper &Hendery (2014: 472).

6.8.26 Western Pantar (lamm1241), TNG, Alor-Pantar

(110) *[[Tabang alaku Duinni Maggangkala]$_{NP}$ [ging]$_{NP}$]$_{NP}$ a-raung yattu*
slave two Duinni Maggangkala 3pl.act INCP-climb tree
ga-ung misingup.
3sg-head sit
'The two slaves Duinni Maggangkala climbed up and sat in the tree.'
(Holton 2014: 53, (105))

Holton (2014: 53, fn. 3) notes that "Duinni Maggagkala is a single (binomial) name give [sic] to the pair of slaves together."

See Holton (2014: 53f.).

6.9 Sino-Tibetan languages

6.9.1 East Geshiza (gesh1238), Burmo-Qiangic

(111) *rdʑæ lmæ=ɲə=t^{h}ə t^{h}ə mp^{h}ri v-s^{h}æ=bɔ, rdʑæ.*
Chinese 3=pl.abs=top redup snake.abs inv-kill.npst.3=mod Chinese.abs
bæ ŋæ=ɲə=t^{h}ə mp^{h}ri mi-s^{h}oŋ.
Tibetan 1=pl.abs=top snake.abs neg-kill.npst.1pl
'The (Han) Chinese, they kill snakes. [...] We Tibetans do not kill snakes.'
(Honkasalo 2019: 480, (7.48))

See Honkasalo (2019: 388, 400, 438, 480, 507, 646) for further examples.

6.9.2 Mandarin (mand1415), Chinese

(112) *congming de ni-men ziji xiang banfa jiejue ba!*
smart LNK you-PL yourselves think ways solve EXCLAM
'you smart people think of a way to solve it yourselves.'
(after Bošković &Hsieh 2013: (53a))

See Bošković &Hsieh (2013: sec. 7.3) for further examples.

6.10 Uralic languages

6.10.1 Hungarian (hung1274), Ugric

(113) *Ti orvos-ok sok-at dolgoz-tok.*
you.PL doctor-PL much-ACC work-INDF.2PL
'You doctors work a lot.'
(Kenesei et al. 1998: 269, (492))

See Kenesei et al. (1998: 269) and Höhn 2016: 559.

6.10.2 Finnish (finn1318), Finnic

(114) *Me naiset menemme nyt saunaan.*
we woman-PL go-1PL now sauna-ILL
'We women will go to the sauna now.'
(Sulkala &Karjalainen 1992: 277, (1335))

See also Höhn (2020: 24f.).

6.11 Creoles

6.11.1 Ndyuka (ndyu1242), Creoles

(115) *u gaanman fu den liba*
1/2PL chief for the-PL river
'you chiefs of the rivers'
(Huttar &Huttar 1994: 467, (2075))

See Huttar &Huttar (1994: 224, 460, 466f.).

6.11.2 Nigerian Pidgin (nige1257), Creoles

(116) *Unà onyìbo pipul no dè chu kola àtộl.*
you.PL white people NEG IPFV chew kola NEG.EMPH
'You white people don't chew kola nut at all.'
(after Faraclas 1996: 181, (802))

See Faraclas (1996: 178, 181).

6.11.3 Kristang (mala1533), Creoles

(117) *kora jenti muré, tudu nus kristáng bai*
when person die all 1PL go
'When people die, all we Kristangs go (to the wake).'
(Baxter 1988: 86, (11))

See Baxter (1988: 86).

6.12 Various

6.12.1 Mi'kmaq (mikm1235), Algonquian

(118) *ninen elnui-yek*
we.EXCL people-1PL.EXCL
'we First Nation people'
(Pacifique et al. 1990: 188)

See Pacifique et al. (1990: 188) for some further (unglossed) examples.

6.12.2 Katu (nucl1297), Katuic

(119) a. *manuih yi* *[Katu]*
people we

b. *yi manuih*
we people
'we people'

c. *yi adi anó yi*
we older.brother younger.brother we
'we older and younger brothers'
Costello 1969: 28, (35–37)

See Costello (1969: 28).

6.12.3 Wari'(wari1268), Chapakuran

(120) *Wirico Mon' co pa' na mijac*
emph:3sg.m m:name infl:m/f.real.pst/prs kill 3sg:real.pst/prs pig
'It was Mon' who killed a pig.'
(after Everett &Kern 1997: 303, (570a))

See Everett &Kern (1997: 303, 310).

6.12.4 Chitimacha (chit1248), Chitimacha

(121) ʔuš panš' ha hananki' namkinada'
'We people who live in this house.'
(Swadesh 1967: 333)

See Swadesh (1967: 333).

6.12.5 Lezgian (lezg1247), Lezgic

(122) *Ča-z čuban-r.i-z, wun har näni-q^h k'wal.i-z*
we-dat shepherd-dat [you:abs every night-postess house-dat
q^{h}ifi-zwazj-di q^{h}sam či-zwa
return-ipfv-ptcp-nmlz] good know-ipfv
'We shepherds know well that you go home every night.'
(after Haspelmath 1993: 259, (682a))

See Haspelmath (1993: 259).

6.12.6 Abkhaz (abkh1244), Northwest Caucasian

(123)

ħ*a(rà)*	*(š^{o} a(rà),*	*darà)*	*a-bàħč-aa-ja-y^{o}-c^{o} a*
we	you	they	ART-garden-PREV-tend-A-PL

‘we (you, they) gardeners’
(Hewitt 1989: 159)

See Hewitt (1989: 157, 159).

6.12.7 Basque (basq1248), Basque

(124) a.

Galdu	*didazue*	***aita-seme-ok***
spoil	3SG.ABS.AUX.1SG.DAT.2PL.ERG	father-son-PRXART.PL.ERG

afari-ta-ko	*gogo*	*guzti-a.*
dinner-LOC-LNK	appetite	all-DET.ABS

‘You, father and son, have spoiled my whole appetite for dinner.’
(de Rijk 2008: 502, (90a))

b.

Zor	*berri-a*	*dugu*	***euskaldun-ok***	*Orixe-rekin.*
debt	new-DET.ABS	3SG.ABS.AUX.1PL.ERG	Basque-PRXART.PL	Orixe-COM

‘We Basques have a new debt to Orixe.’
(de Rijk 2008: 502, (91a))

See Saltarelli (1988: 210), Trask (2003: 122), de Rijk (2008: 482, 501f.), Areta (2009: 67) and especially Artiagoitia (2012).

6.12.8 Classical Nahuatl (clas1250), Aztecan

(125) a.

***Ni**cuīca*	***ni**Petoloh.*
I-sing	I-am-Peter

‘I, Peter, sing.’

b.

***Nēch**itta*	***ni**Petoloh.*
he-sees-me	I-am-Peter

‘He sees me, Peter.’
(Andrews 1975: 193)

c.

***No**cal*	***ni**Petoloh.*
It-is-my-house/they-are-my-houses	I-am-Peter

‘It is my house (and I am Peter)./They are my houses (and I am Peter).’
(Andrews 1975: 194)

See Andrews (1975: 192–194) and Andrews 2003: ch. 17.3.

6.12.9 Evenki (even1259), Tungusic

(126) *Bu bejumimni-l eme-re-∅.*
we hunter-PL come-NFUT-3PL
'We, hunters, came.'
(Nedjalkov 1997: 199, (794))

See Nedjalkov (1997: 197, 199).

6.12.10 Hixkaryana (hixk1239), Cariban

(127) a. *mɨnayarɨ horɨ amna ntono. nɨmno* *Hixkaryana*
species.of.leaf seeking we.EXCL went house

hokono rma amna
one.occupied.with same-ref we.EXCL

'We housebuilders went looking for leaves'
(Derbyshire 1979: 131, (290))

b. *nux mokro raheno*
my.younger.brother that.one he.seduced.me
'That younger brother of mine seduced me'
(Derbyshire 1979: 132, (293a))

Derbyshire (1979: 131) suggests that Hixkaryana does not have integrated adnominal person marking (and no adnominal demonstratives).

6.12.11 Japanese (nucl1643), Japanese

(128) *wareware nihonjin*
we Japanese
'we Japanese'
(Hinds 1988: 254)

See Hinds (1988: 254, 261) and for more detailed discussion with different theoretical proposals: Noguchi (1997: 780), Furuya (2008: sec. 3.2) and Inokuma (2009).

6.12.12 Korean (kore1280), Korean

(129) wuli-(tul) hankwuk salam
‘we Koreans’
(Sohn 1994: 292)

(130) a. *wuli ttokttokhan enehakcatul*
we smart linguists

b. *ttokttokhan wuli enehakcatul*
smart we linguists
‘we smart linguists’
(Choi 2014: 151, (15))

See Sohn (1994: 284; 292) and Choi (2014: 151–154).

6.12.13 Kalaallisut/West Greenlandic (kala1399), Eskimo

(131) a. *kalaalli-t* ***uagut***
Greenlander-ABS.PL we
‘we Greenlanders’
(after Fortescue 1984: 110; gloss extrapolated)

b. ***uagut*** *kalaali-u-sugut*
we Greenlander-be-1PL.PTCP
‘we Greenlanders’
(after Fortescue 1984: 257)

See Fortescue (1984: 110, 253, 256f.).

6.12.14 Lakkia (lakk1238), Kadai

(132) *tau*^{51} *hou*^{24} *ʔat*^{55} *jen*^{11} *kjã:u*^{24}
1PL two sister
‘we, the two sisters’
(Fan 2019: 137, (40))

6.12.15 Turkish (nucl1301), Turkic

(133) *biz Türk-ler vatan-ımız-ı sev-er-iz*
we Turk-PL mother.country-1PL-ACC love-AOR-1PL
'We Turks love our country'
(Kornfilt 1997: 297, (1075))

See Kornfilt (1997: 288, 297f.).

Abbreviations

A = agent, ABS = absolutive, ACC = accusative, ACT = actor, ADDR = addressee, AIC = Akike information criterion, ANAPH = anaphoric pronoun, AOR = aorist, APC = adnominal pronoun construction, APPL = applicative, ART = article, ASS = assertive, AUX = auxiliary, BPC = bound person construction, CAUS = causative, CERT = certainty of assertion, CF = counterfactual, CG = common gender, CL = clitic, CNT = continuous, COM = comitative, COMPEL = compellative, CONTR = contrastive, DAT = dative, DEF = definite, DEM = demonstrative, DET = determiner, DS = different subject, DSO = dissociative, DU = dual, DUR = durative, EMPH = emphatic, ERG = ergative, EXCL = exclusive, EXCLAM = exclamation, F = feminine, FIN = clause-final verb, FOC = focus, FPRON = focus pronoun, FV = final vowel, GEN = genitive, GENER = generic, GVN = given, HABIT = habitual, IE = Indo-European, ILL = illative, IMP = imperative, INAN = inanimate, INCL = inclusive, IND = indicative, INDF = indefinite, INF = infinitive, INFL = inflection, INV = inverse, IPFV = imperfective, IRR = irrealis, LIG = ligature, LNK = adnominal linker, LOC = locative, M = masculine, MOD = modal discourse enclitic (East Geshiza), N = neuter, NACT = non-actual, NCL = noun class marker, NEG = negative, NF = non-feminine, NFIN = non-final marker, NFUT = non-future, NMLZ = nominalizer, NOM = nominative, NPST = non-past, OBJ = object, OBL = oblique, PASS = passive, $PERS_N$ = adnominal person, PFV = perfective, PL = plural, PN = Pama-Nyungan, POSS = possessive, POSTESS = postessive, PPDC = personal pronoun-demonstrative construction, PREF = prefix, PREV = preverb, PRF = perfect, PROG = progressive, PROH = prohibitive, PROX = proximal, PRS = present, PRTCL = particle, PRXART = proximate article, PST = past, PTCP = participle, PURP = purposive, REAL = realis, REDUP = reduplication, REFL = reflexive, S = argument of intransitive verb, SBJ = subject, SBJV = subjunctive, SG = singular, SOFTMUT = soft mutation, SPEC = specific, SPRON = subject pronoun, SS = same subject, STVZR = stativiser, TAM = tense aspect mood marker, TNG = Trans-New Guinea, TODPST = today's past, TOP = topic, TRI = trial/paucal, U = unmarked, VOC = vocative.

References

Ackema, Peter & Ad Neeleman. 2013. Subset controllers in agreement relations. *Morphology* 23. 291–323.

Aikhenvald, Alexandra Y. 2008. *The Manambu language of East Sepik, Papua New Guinea*. Oxford: Oxford University Press.

Álvarez, Rosario, Henrique Monteagudo & Xosé Luis Regueira. 1986. *Gramática Galega*. Vigo: Editorial Galaxia.

Andrews, J. Richard. 1975. *Introduction to classical Nahuatl*. Austin, London: University of Texas Press.

Andrews, J. Richard. 2003. Introduction to Classical Nahuatl. revised edition. University of Oklahoma Press Norman.

Areta, Mikel Martínez. 2009. The category of number in Basque: I. Synchronic and historical aspects. *Fontes Linguae Vasconum* 110. 63–98.

Artiagoitia, Xabier. 2012. The DP hypothesis in the grammar of Basque. In Urtzi Etxeberria, Ricardo Etxepare & Myriam Uribe-Etxebarria (eds.), *Noun phrases and nominalization in Basque: Syntax and semantics*, 21–77. Amsterdam: John Benjamins.

Asher, R. E.. 1985. *Tamil*. Beckenham: Croom Helm.

Asher, R. E. & T. C. Kumari. 1997. *Malayalam*. London: Routledge.

Ashton, E.O., E.M.K. Mulira, E.G.M. Ndawula & A.N. Tucker. 1954. *A Luganda grammar*. London: Longmans.

Austin, Peter. 1981. *A grammar of Diyari, South Australia*. Cambridge: Cambridge University Press.

Austin, Peter. 2013. *A grammar of Diyari, South Australia*. Cambridge: Cambridge University Press 2nd edn.

Bauer, Winifred. 1993. *Maori*. London: Routledge.

Bauer, Winifred. 1997. *The Reed reference grammar of Māori*. Auckland: Reed Books.

Baxter, Alan N. 1988. *A grammar of Kristang (Malacca Creole Portuguese)* (Pacific Linguistics 95). Canberra: The Australian National University.

Besnier, Niko. 2000. *Tuvaluan*. London: Routledge.

Bhatia, Tej K. 1993. *Punjabi*. London: Routledge.

Borg, Albert & Marie Azzopardi-Alexander. 1997. *Maltese*. London: Routledge.

Boswell, Frederick. 2018. *A grammar of Cheke Holo*: Leiden University dissertation.

Bošković, Željko & I-Ta Chris Hsieh. 2013. On word order, binding relations, and plurality in Chinese noun phrases. *Studies in Polish Linguistics* 8. 173–204.

Bowe, Heather J. 1990. *Categories, constituents and constituent order in Pitjantjatjara. An Aboriginal language of Australia*. London: Routledge.

Bruce, Les. 1984. *The Alamblak language of Papua New Guinea (East Sepik)*. Canberra: The Australian National University.

Böhm, Gerhard. 1985. *Khoe-kowap. Einführung in die Sprache der Hottentotten, Nama-Dialekt*. Wien: Afro-pub.

Cardinaletti, Anna. 1994. On the internal structure of pronominal DPs. *The Linguistic Review* 11. 195–219.

Carlson, Robert. 1994. *A grammar of Supyire*. Berlin: Mouton de Gruyter.

Choi, Jaehoon. 2014. *Pronoun-noun constructions and the syntax of DP*: University of Arizona dissertation.

Comrie, Bernard & Norval Smith. 1977. Lingua descriptive studies: Questionnaire. *Lingua* 42(1). 1–72.

Cornilescu, Alexandra & Alexandru Nicolae. 2014. Classifying pronouns: The view from Romanian. *Bucharest Working Papers in Linguistics* 16(1). 5–33.

Corver, Norbert. 2008. Uniformity and diversity in the syntax of evaluative vocatives. *Journal of Comparative Germanic Linguistics* 11(1). 43–93.

Costello, Nancy A. 1969. The Katu noun phrase. *Mon-Khmer Studies* 3. 21–35.

Coulmas, Florian. 1982. Some remarks on Japanese deictics. In Jürgen Weissenborn & Wolfgang Klein (eds.), *Here and there. Cross-linguistic studies on deixis and demonstration*, 209–221. Amsterdam: John Benjamins.

Crane, Thera M., Larry M. Hyman & Simon Nsielanga Tukumu. 2011. *A grammar of Nzadi [b.865]. A Bantu language of the Democratic Republic of the Congo*, vol. 147 University of California Publications in Linguistics. Berkeley & Los Angeles: University of California Press.

Davies, John. 1989. *Kobon*. Croom Helm. Reprint of 1981 first edition.

Davis, Karen. 2003. *A grammar of the Hoava language, Western Solomons*. Canberra: The Australian National University.

de Bruyne, Jacques. 1995. *A comprehensive Spanish grammar*. Malden (MA), Oxford: Blackwell. Adapted with additional material by Christopher J. Pountain.

de Rijk, Rudolf P.G. 2008. *Standard Basque: A progressive grammar*. Cambridge (MA): MIT Press.

Delorme, Evelyn & Ray C. Dougherty. 1972. Appositive NP constructions. *Foundations of Language* 8. 2–29.

Derbyshire, Desmond C. 1979. *Hixkaryana*. Amsterdam: North-Holland Publishing Company.

D'Jernes, Lucille S. 2002. Arop-lokep. In John Lynch, Malcolm Ross & Terry Crowley (eds.), *The oceanic languages* Curzon Language Family Series, 249–269. Richmond: Curzon.

Dol, Philomena. 2007. *A grammar of Maybrat. A language of the Bird's Peninsula, Papua Province, Indonesia*, vol. 586 Pacific Linguistics. Canberra: Pacific Linguistics.

Doss, Madiha. 1979. The position of the demonstrative *da*, *di* in Egyptian Arabic: a diachronic inquiry. *Annales islamologiques* 15. 349–357.

Dryer, Matthew S. 1989. Large linguistic areas and language sampling. *Studies in Language* 13(2). 257–292.

Dryer, Matthew S. 2013a. Order of adposition and noun phrase (v2020.3). In Matthew S. Dryer & Martin Haspelmath (eds.), *The World Atlas of Language Structures Online*, Zenodo.

Dryer, Matthew S. 2013b. Order of demonstrative and noun (v2020.3). In Matthew S. Dryer & Martin Haspelmath (eds.), *The World Atlas of Language Structures Online*, Zenodo.

Dryer, Matthew S. 2013c. Order of genitive and noun (v2020.3). In Matthew S. Dryer & Martin Haspelmath (eds.), *The World Atlas of Language Structures Online*, Zenodo.

Dryer, Matthew S. 2013d. Order of object and verb (v2020.3). In Matthew S. Dryer & Martin Haspelmath (eds.), *The World Atlas of Language Structures Online*, Zenodo.

Dryer, Matthew S. & Martin Haspelmath (eds.). 2013. *The World Atlas of Language Structures Online*. Leipzig: Max Planck Institute for Evolutionary Anthropology.

Evans, Nicholas D. 1995. *A grammar of Kayardild*. Berlin: Mouton de Gruyter.

Everett, Dan & Barbara Kern. 1997. *Wari'*. London: Routledge.

Fan, Wenjia. 2019. *A grammar of Lakkja, South China*. Melbourne: The University of Melbourne dissertation.

Faraclas, Nicholas G. 1996. *Nigerian Pidgin*. London: Routledge.

Feldman, Harry. 1986. *A grammar of Awtuw* (Pacific Linguistics 94). Canberra: The Australian National University.

Fortescue, Michael. 1984. *West Greenlandic*. Beckenham: Croom Helm.

Frajzyngier, Zygmunt. 1993. *A grammar of Mupun*. Berlin: Dietrich Reimer Verlag.

Furuya, Kaori. 2008. DP hypothesis for Japanese “bare” noun phrases. In Łukasz Abramowicz, Stefanie Brody, Toni Cook, Ariel Diertani, Aviad Eilam, Keelan Evanini, Kyle Gorman, Laurel MacKenzie & Joshua Tauberer (eds.), *Proceedings of the 31st Annual Penn Linguistics Colloquium*, vol. 14 U. Penn Working Papers in Linguistics, 149–162. Philadelphia: University of Pennsylvania.

Gary, Judith Olmsted & Gamal-Eldin. 1982. *Cairene Egyptian Colloquial Arabic*. London: Croom Helm.

Gasser, Emily Anne. 2014. *Windesi Wamesa morphophonology*: Yale University dissertation.

Gravelle, Gloria J. 2010. *A grammar of Moskona: An East Bird's Head Language of West Papua, Indonesia*. Amsterdam: Vrije Universiteit Amsterdam dissertation.

Haacke, Wilfrid H. G. 2013. Morphology. Namibian Khokoe (Nama/Damara). In Rainer Vossen (ed.), *The Khoesan languages*, 141–151. London: Routledge.

Haacke, Wilfrid Heinrich Gerhard. 1976. *A Nama grammar: the noun-phrase*. University of Cape Town MA thesis.

Haacke, Wilfrid Heinrich Gerhard. 1977. The so-called “personal pronoun” in Nama. In Anthony Traill (ed.), *Khoisan linguistic studies 3*, 43–62. Johannesburg: African Studies Institute.

Haiman, John. 1980. *Hua: A Papuan language of the Eastern Highlands of New Guinea*. Amsterdam: John Benjamins.

Hale, Ken. 1973. Person marking in Warlpiri. In Stephen R. Anderson & Paul Kiparsky (eds.), *Festschrift for Morris Halle*, 308–344. New York: Holt, Rinehart and Winston.

Hamel, Patricia J. 1994. *A grammar and lexicon of Loniu, Papua New Guinea* (Pacific Linguistics 103). Canberra: The Australian National University.

Hammarström, Harald, Robert Forkel, Martin Haspelmath & Sebastian Bank. 2021. Glottolog 4.5. Max Planck Institute for the Science of Human History.

Harvey, Andrew. 2018. *The Gorwaa noun: Toward a description of the Gorwaa language*. London: SOAS dissertation.

Haspelmath, Martin. 1993. *A grammar of Lezgian*. Berlin: Mouton de Gruyter.

Haviland, John. 1979. Guugu Yimidhirr. Sketch grammar. In Robert M.W. Dixon & Barry Biake (eds.), *Handbook of Australian languages*, vol. 1, 26–180. The Australian National University.

Hemmilä, Ritva & Pirkko Luoma. 1987. Urim grammar. Obtained from https://www.sil.org/resources/archives/31244 on 4/4/2020.

Hewitt, B. George. 1989. *Abkhaz*. London: Routledge. First published in 1979 by North-Holland Publishing Company.

Hijmans, Robert J. 2022. *geosphere: Spherical trigonometry*. R package version 1.5-18.

Himmelmann, Nikolaus P. 1997. *Deiktikon, Artikel, Nominalphrase: Zur Emergenz synaktischer Struktur*. Tübingen: Niemeyer.

Hinds, John. 1988. *Japanese*. London: Routledge. First published in 1986 by Croom Helm.

Holes, Clive. 1990. *Gulf Arabic*. London: Croom Helm.

Holton, Gary. 2014. Western Pantar. In Antoinette Schapper (ed.), *The Papuan languages of Timor, Alor and Pantar: Volume 1. Sketch grammars*, 23–95. Berlin: Mouton de Gruyter.

Honeyman, Thomas. 2016. *A grammar of Momu, a language of Papua New Guinea*. Canberra: The Australian National University dissertation.

Honkasalo, Sami. 2019. *A grammar of Eastern Geshiza. A culturally anchored description*. Helsinki: University of Helsinki dissertation.

Hualde, José Ignacio. 1992. *Catalan*. London: Routledge.

Huang, C.-T. James, Y.-H. Audrey Li & Yafei Li. 2009. *The syntax of Chinese*. Cambridge: Cambridge University Press.

Hurtado, Alfredo. 1985. The unagreement hypothesis. In L. King & C. Maley (eds.), *Selected papers from the thirteenth linguistic symposium on Romance languages*, 187–211. Amsterdam: John Benjamins.

Huttar, George L. & Mary L. Huttar. 1994. *Ndyuka*. London: Routledge.

Höhn, Georg F. K. 2016. Unagreement is an illusion: Apparent person mismatches and nominal structure. *Natural Language and Linguistic Theory* 34(2). 543–592.

Höhn, Georg F. K. 2017. *Non-possessive person in the nominal domain*: University of Cambridge dissertation.

Höhn, Georg F. K. 2020. The third person gap in adnominal pronoun constructions. *Glossa* 5(1). 69.

Höhn, Georg F. K., Giuseppina Silvestri & M. Olimpia Squillaci. 2016. Unagreement between Italian and southern Italian dialects. *Rivista di Grammatica Generativa* 38. 137–147.

Höhn, Georg F. K., Giuseppina Silvestri & M. Olimpia Squillaci. 2017. Greek and Romance unagreement in Calabria. *Journal of Greek Linguistics* 17(2). 263–292.

Inokuma, Sakumi. 2009. So-called pronoun-noun construction in Japanese: A new perspective on nominal syntax. *Linguistic Research* 25. 31–42.

Jaggar, Philip J. 2001. *Hausa*. Amsterdam, Philadelphia (PA): John Benjamins.

Johannessen, Janne Bondi. 2008. The pronominal psychological demonstrative in Scandinavian: Its syntax, semantics and pragmatics. *Nordic Journal of Linguistics* 31(2). 161–192.

Julien, Marit. 2005. *Nominal phrases from a Scandinavian perspective*. Amsterdam: John Benjamins.

Keesing, Roger M. 1985. *Kwaio grammar* (Pacific Linguistics 88). Canberra: The Australian National University.

Keizer, Evelien. 2016. We teachers, you fools: Pro+N(P) constructions in Functional Discourse Grammar. *Language Sciences* 53. 177–192.

Kenesei, István, Robert M. Vago & Anna Fenyvesi. 1998. *Hungarian*. London: Routledge.

Kilian-Hatz, Christa. 2008. *A grammar of modern Khwe*. Köln: Rüdiger Köppe.

Klamer, Marian. 2014. Kaera. In Antoinette Schapper (ed.), *The Papuan languages of Timor, Alor and Pantar: Volume 1. Sketch grammars*, 97–146. Berlin: Mouton de Gruyter.

Kluge, Angela. 2017. *A grammar of Papuan Malay*. Berlin: Language Science Press.

Kornfilt, Jaklin. 1997. *Turkish*. London: Routledge.

Kratochvíl, František. 2014. Sawila. In Antoinette Schapper (ed.), *The Papuan languages of Timor, Alor and Pantar: Volume 1. Sketch grammars*, 351–438. Berlin: Mouton de Gruyter.

Köhler, Oswin. 1989. *Die Welt der Kxoé-Buschleute im südlichen Afrika: Eine Selbstdarstellung in ihrer eigenen Sprache*, vol. Vol. 1: Die Kxoé-Buschleute und ihre ethnische Umgebung. Berlin: Dietrich Reimer Verlag.

Lawrenz, Birgit. 1993. *Apposition. Begriffsbestimmung und syntaktischer Status*. Tübingen: Narr.

Lekakou, Marika & Kriszta Szendrői. 2012. Polydefinites in Greek: Ellipsis, close apposition and expletive determiners. *Journal of Linguistics* 48. 107–149.

Lyons, Christopher. 1999. *Definiteness*. Cambridge: Cambridge University Press.

Maho, Jouni F. 1998. *Few people, many tongues*. Windhoek: Gamsberg Macmillan.

Mahootian, Shahrzad. 1997. *Persian*. London: Routledge.

Mallinson, Graham. 1986. *Rumanian*. London: Croom Helm.

Merlan, Francesca. 1989. *Mangarayi*. London: Croom Helm. Reprint of 1982 North-Holland Publishing edition.

Mpiranya, Fidèle. 2015. *Swahili grammar and workbook*. New York: Routledge.

Nedjalkov, Igor. 1997. *Evenki*. London: Routledge.

Newman, Paul. 2000. *The Hausa language. An encyclopedic reference grammar*. New Haven, London: Yale University Press.

Noguchi, Tohru. 1997. Two types of pronouns and variable binding. *Language* 73. 770–797.

Norman, Boris. 2001. Субстантивное подлежащее при глаголах в 1-м лице множественного числа в болгарском языке (Двама студенти търсим работа) [substantivnoe podležaščee pri glagolax v 1-m lice množestvennogo čisla v bol'garskom jazyke (*Dvama studenti tərsim rabota*)]. In Alexander Kiklevič (ed.), *Количественность и градуальность. Quantität und Graduierung in der natürlichen Sprache* (Die Welt der Slaven 11), 77–86. München: Otto Sagner.

Obata, Kazuko. 2003. *A grammar of Bilua. A Papuan language of the Solomon Islands* (Pacific Linguistics 540). Canberra: The Australian National University.

Osenova, Petya. 2003. On subject-verb agreement in Bulgarian (an HPSG-based account). In Peter Kosta, Joanna Błaszczak, Jens Frasek, Ljudmila Geist & Marzena Żygis (eds.), *Investigations into formal Slavic linguistics. Contributions of the fourth European conference on formal description of Slavic languages – FDSL IV*, 661–672. Frankfurt/Main: Peter Lang.

Pacifique, Father, John Hewson & Bernard Francis. 1990. *The Micmac grammar of Father Pacifique*, vol. 7 Memoir. Winnipeg: Algonquian and Iroquoian Linguistics.

Palmer, Bill. 2008. *Kokota grammar* (Oceanic Linguistics Special Publication 35). Honolulu: University of Hawai'i Press.

Palmer, Bill. 2017. Categorial flexibility as an artefact of the analysis. Pronouns, articles and the DP in Hoava and Standard Fijian. *Studies in Language* 41(2). 408–444.

Pandharipande, Raeshwari V. 1997. *Marathi.* London: Routledge.

Papadimitriou, Panagiotis. 2008. *Τα Πομάκικα. Συγχρονική περιγραφή μιας νότιας τοπικής ποικιλίας της Αναλυτικής Σλαβικής από τη Μύκη του Νομού Ξάνθης* [Ta pomakika. Sygxroniki perigrafi mias notias topikis poikilias tis Analytikis Slavikis apo ti Myki tou Nomou Ksanthis]. Thessaloniki: Adelfon Kyriakidi.

Patz, Elisabeth. 2002. *A grammar of the Kuku Yalanji language of North Queensland* (Pacific Linguistics 527). Canberra: The Australian National University.

Paul, Ileana & Lisa deMena Travis. 2019. Augmented pronoun constructions in Malagasy across time and space. In Eszter Ronai, Laura Stigliano & Yenen Sun (eds.), *Proceedings of the fifthy-fourth annual meeting of the Chicago Linguistic Society (cls 54)*, 409–423. ¢hicago: The Chicago Linguistic Society.

Pesetsky, David. 1978. Category switching and so-called so-called pronouns. In Donka Farkas, Wesley M. Jacobsen & Karol W. Todrys (eds.), *Chicago linguistic society*, vol. 14, 350–360. Chicago.

Postal, Paul. 1969. On so-called "pronouns" in English. In David A. Reibel & Sanford A. Schane (eds.), *Modern studies in English: Readings in Transformational Grammar*, 201–226. Englewood Cliffs (New Jersey): Prentice Hall.

Rauh, Gisa. 2003. Warum wir Linguisten "euch Linguisten", aber nicht "sie Linguisten" akzeptieren können. Eine personendeiktische Erklärung. *Linguistische Berichte* 196. 390–424.

Rauh, Gisa. 2004. Warum 'Linguist' in 'ich/du Linguist' kein Schimpfwort sein muß. Eine konversationstheoretische Erklärung. *Linguistische Berichte* 197. 77–105.

Reece, Laurie. 1970. *Grammar of the Wailbri language of central Australia* (Oceania Linguistic Monographs 13). Sydney: University of Sydney, Australia.

Reesink, Ger P. 1987. *Structures and their functions in Usan. A Papuan language of Papua New Guinea.* Amsterdam: John Benjamins.

Reesink, Ger P. 1999. *A grammar of Hatam. Bird's Head peninsula, Irian Jaya* (Pacific Linguistics C-146). Canberra: The Australian National University.

Reesink, Ger P. 2002. A grammar sketch of Sougb. In Ger P. Reesink (ed.), *Languages of the eastern Bird's Head* (Pacific Linguistics 524), chap. 4, 181–275. Canberra: The Australian National University.

Renck, G. L. 1975. *A grammar of Yagaria* (Pacific Linguistics 4). Canberra: The Australian National University.

Rennison, John R. 1997. *Koromfe*. London: Routledge.

Roberts, John R. 1987. *Amele*. London: Croom Helm.

Robinson, Laura C. & John W. Haan. 2014. Adang. In Antoinette Schapper (ed.), *The Papuan languages of Timor, Alor and Pantar: Volume 1. Sketch grammars*, 221–283. Berlin: Mouton de Gruyter.

Roehrs, Dorian. 2005. Pronouns are determiners after all. In Marcel den Dikken & Christina M. Tortora (eds.), *The function of function words and functional categories*, 251–285. Amsterdam: John Benjamins.

Round, Erich R. 2013. *Kayardild morphology and syntax*. Oxford: Oxford University Press.

Rust, Friederich. 1965. *Praktische Namagrammatik*. Cape Town: A.A. Balkema.

Rutkowski, Pawel. 2002. Noun/pronoun asymmetries: evidence in support of the DP hypothesis in Polish. *Jezikoslovlje* 3(1–2). 159–170.

Saltarelli, Mario. 1988. *Basque*. London: Croom Helm.

Schapper, Antoinette. 2014. Kamang. In Antoinette Schapper (ed.), *The Papuan languages of Timor, Alor and Pantar: Volume 1. Sketch grammars*, 285–349. Berlin: Mouton de Gruyter.

Schapper, Antoinette & Rachel Hendery. 2014. Wersing. In Antoinette Schapper (ed.), *The Papuan languages of Timor, Alor and Pantar: Volume 1. Sketch grammars*, 439–503. Berlin: Mouton de Gruyter.

Schaub, Willi. 1985. *Babungo*. London: Croom Helm.

Schröter, Andrea. 2021. Adnominale Pronominalkonstruktionen im Dänischen: eine empirische Studie. BA thesis, University of Göttingen.

Scott, Graham. 1978. *The Fore language of Papua New Guinea*. Canberra: The Australian National University.

Seiler, Walter. 1985. *Imonda, a Papuan language* (Pacific Linguistics 93). Canberra: The Australian National University.

Sigurðsson, Halldór Ármann & Jim Wood. 2020. "We Olaf": Pro[(x-)NP] constructions in Icelandic and beyond. *Glossa* 5(1). 16.

Sneddon, James Neil. 1996. *Indonesian. A comprehensive grammar*. London: Routledge.

Sohn, Ho-Min. 1994. *Korean*. London: Routledge.

Sommerstein, Alan H. 1972. On the so-called definite article in English. *Linguistic Inquiry* 3. 197–209.

Sridhar, S. N. 1990. *Kannada*. London: Routledge.

Stavrou, Melita. 1995. Epexegesis vs. apposition in Modern Greek. In *Scientific bulletin of the School of Philology*, vol. 5, 217–250. Thessaloniki: Aristotle University.

Sulkala, Helena & Merja Karjalainen. 1992. *Finnish*. London: Routledge.

Swadesh, Morris. 1967. Chitimacha. In Cornelius Osgood (ed.), *Linguistic structures of native America*, 312–336. New York: Johnson Reprint Corporation.

Tayebwa, Daphine Doreen. 2014. *Demonstrative determiners in Runyakore-Rukiga*. Trondheim Norwegian University of Science and Technology MA thesis. Available at www.diva-portal.org/smash/get/diva2:743912/FULLTEXT01.pdf, accessed 7 January 2017.

Taylor, Charles. 1985. *Nkore-Kiga*. London: Croom Helm.

Terrill, Angela. 2003. *A grammar of Lavukaleve*. Berlin: Mouton de Gruyter.

Terrill, Angela. 2011. Languages in contact: An exploration of stability and change in the Solomon Islands. *Oceanic Linguistics* 50(2). 312–337.

Trask, R.L. 2003. The noun phrase: nouns, determiners and modifiers; pronouns and names. In José Ignacio Hualde & Jon Ortiz de Urbina (eds.), *A grammar of Basque*, 113–170. Berlin, New York: Mouton de Gruyter.

Treis, Yvonne. 2008. *A grammar of Kambaata (Ethiopia). Part 1: Phonology, nominal morphology, and non-verbal predication.* Köln: Rüdiger Köppe.

van der Wal, Jenneke. 2022. *A featural typology of Bantu agreement.* Oxford, New York: Oxford University Press.

Wali, Kashi & Omkar N. Koul. 1997. *Kashmiri.* London: Routledge.

Wegener, Claudia. 2012. *A grammar of Savosavo.* Berlin: Mouton de Gruyter.

Whitehead, Carl R. 2006. *A reference grammar of Menya, an Angan language of Papua New Guinea.* Winnipeg: University of Manitoba dissertation. Slightly revised web-version of 2004 thesis. Available on http://citeseerx.ist.psu.edu/viewdoc/download?doi=10.1.1.476.9453&rep=rep1&type=pdf, accessed 12/4/2020.

Whitehead, Carl R. 2013. Pronominal systems in Menya, with a preliminary look at related languages. *Language and Linguistics in Oceania* 5. 1–32.
